# Cost-Effective Multi-Channel MolOrbImage for Machine-Learned Excited-State Properties of Practical Photofunctional Materials

**DOI:** 10.1021/acs.jctc.5c01721

**Published:** 2026-01-26

**Authors:** Ziyong Chen, Jonathan Lam, Vivian Wing-Wah Yam

**Affiliations:** † Institute of Molecular Functional Materials and Department of Chemistry, 25809 The University of Hong Kong, Hong Kong 999077, P. R. China; ‡ Hong Kong Quantum AI Lab Limited, Hong Kong 999077, P. R. China

## Abstract

Leveraging our recent development, which incorporates hole and particle information into the multi-channel molecular orbital image (MolOrbImage), to generate exceptional accuracy (mean absolute error, MAE < 0.1 eV) in predicting excited-state energies of practical photofunctional materials containing several hundred atoms, we have advanced the implementation of a new approach to overcome the high computational cost of mean-field ground-state calculations that limits its application in high-throughput materials discovery. In this work, low-cost approaches for generating approximate orbitals, including the superposition of atomic densities technique and the semiempirical tight-binding method, have been employed to construct cost-effective multi-channel MolOrbImages. By connecting with a convolutional neural network, the performance of our model is evaluated for both small organic molecules (MAE < 0.1 eV) and practical photofunctional materials (MAE < 0.14 eV). Perturbation analysis of MolOrbImages highlights the importance of frontier orbital energies, which further motivates the adoption of transfer learning techniques to reduce prediction errors in excited-state energies.

## Graphical abstract

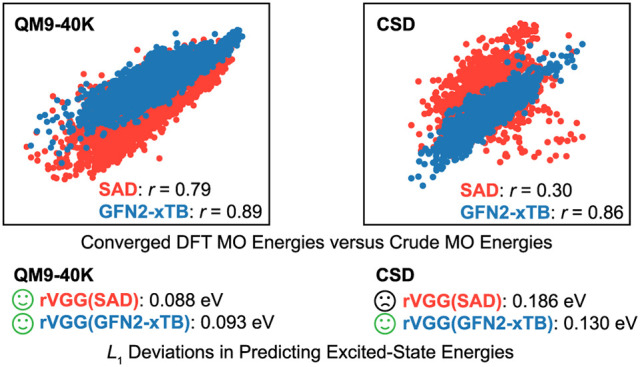

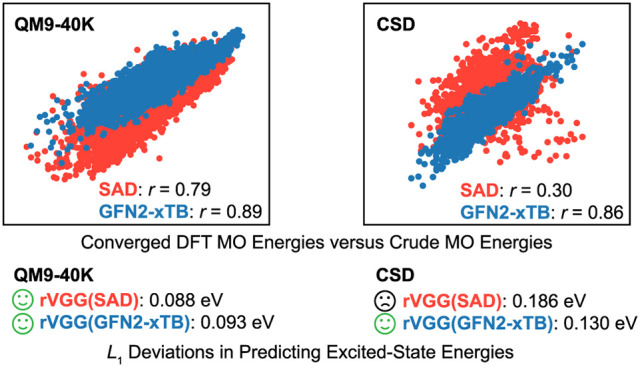

## 1 Introduction

The parallel understanding and manipulation of molecules in their excited states are crucial for the rational design of novel materials with tailored photofunctionalities.^1^ Excited-state quantum chemical calculations, which are based on methods such as time-dependent density functional theory (TDDFT),^2^ second-order algebraic diagrammatic construction (ADC2),^3^ and density matrix renormalization group,^4^ provide insights into the energy landscape beyond the molecular ground state (S_0_). These calculations have been applied to elucidate the origins of electronic transitions in the open and closed forms of photochromic diarylethenes,^5–7^ to quantify the magnitudes of singlet–triplet $\left(\Delta E_{S_{1}-T_{1}}\right)$ and triplet–triplet $\left(\Delta E_{T_{2}-T_{1}}\right)$ energy gaps in thermally activated delayed fluorescence (TADF)^8–11^ and thermally stimulated delayed phosphorescence (TSDP)^11–14^ emitters, respectively, and to evaluate the circular dichroism spectral profiles of [*n*]­cycloparaphenylene-pillar­[5]­arene bismacrocycles.^15^ Consequently, theoretical calculations underpin our fundamental understanding of photophysical properties and guide the rational design and synthesis of new materials based on domain knowledge. However, data-driven attempts, including enumeration of core structures substituted with various functional spacers and terminal moieties,^16–18^ similarity-controlled manipulation of molecular string representations,^19^ deep neural network (NN)-enhanced genetic algorithms,^20^ and denoising diffusion probabilistic models,^21^ can generate a vast number of candidate molecules beyond the capacity of traditional quantum chemical calculations for high-throughput virtual screening (HTVS). As such, there is a pressing need to develop robust, accurate, and cost-effective simulation protocols. Excited-state machine learning (ML) models^22–24^ have emerged as promising and computationally feasible tools to address this challenge.

There remains substantial potential for developing ML models that can be applied across the diverse chemical space of practically relevant molecules.^25–27^ Utilizing sorted Coulomb matrix (CM) descriptors, the kernel ridge regression model achieves a mean absolute error (MAE) of approximately 0.4 eV against the CC2/def2-TZVP (the second-order approximate coupled cluster theory) computed $E_{S_{1}}$ within the QM9 ^28^ data set.^29^ The number of training molecules (*N*_train_) is 10,000. Incorporating the $E_{S_{1}}$ values computed at the TDDFT/PBE0/def2-TZVP level into the $\Delta_{\mathrm{TDDFT}}^{\mathrm{CC}2}$ -ML framework markedly reduces the prediction error to 0.08 eV. Further study on the same data set using the SchNet model demonstrates a MAE of 0.494 eV for $E_{S_{1}}$ at the PBE0/def2-TZVP level.^30^ More advanced NN architectures, such as the convolutional NN (CNN) with CM descriptors and the deep tensor NN (DTNN) utilizing atomic charges and interatomic distances, achieve root-mean-square errors (RMSEs) of 0.304 and 0.251 eV, respectively,^31^ for excitation energies of S_1_–S_16_ states within combined QM7b^32^ and QM9 data sets (*N*_train_ = 6,000). Increasing the size of the training set to 132,000 molecules further reduces RMSEs to 0.251 eV for CNN and 0.186 eV for DTNN. These findings indicate that excited-state ML models using only the geometric structure information tend to exhibit relatively large prediction errors at small *N*_train_, with error reduction progressing slowly as *N*_train_ increases, even for small organic molecules.

In this regard, integrating electronic structure information can accelerate the convergence of prediction accuracy with respect to *N*_train_ and improve the generalization ability of ML models. For example, in predicting the transition dipole moment (TDM) of *N*-methylacetamide, replacing atomic charges in the CM descriptor with natural population analysis (NPA) charges significantly enhances prediction accuracy for the *n* → π* TDM, with the Pearson correlation coefficient (*r*) increasing from 0.39 to 0.84.^33^ Other illustrative approaches include the spectrum of approximated Hamiltonian matrices based on atomic- and bond-density-derived representations [SPA^H^M­(a) and SPA^H^M­(b)],^34^ which learn hole and particle densities of the π → π* transition using quantum chemical descriptors derived from the Laikov–Briling guess densities.^35^ The molecular orbital-based (MOB) Fock–Coulomb–Exchange (FJK) representation has been developed to predict $E_{S_{1}}$ energies for the QM7b data set, achieving a MAE of 0.18 eV with *N*_train_ = 5,000.^36^ Additionally, graph NNs combined with extended tight-binding-based simplified Tamm–Dancoff approximation^37^ (xTB-sTDA) calibrated against TDDFT and CC2 computed excited-state energies yield a MAE of around 0.14 eV.^38^ Other relevant quantum chemically informed ML models include MOB-ML^39,40^ and OrbNet-Equi,^41^ which are primarily focused on high-level S_0_ properties.

Inspired by the success of deep CNNs in computer vision, we recently reported a series of quantum descriptors based on MO images (MolOrbImage). The proof-of-principle study involves a vector of Hartree–Fock computed MO energies (ϵ) as well as a one-channel MolOrbImage of electron repulsion integrals (ERIs) as input features.^42^ When combined with the MO-NN model, testing on a subset of QM9 (QM9-40K, *N*_train_ = 32,000) yields MAEs of 0.140 and 0.105 eV for energies of the lowest-lying S_1_ and T_1_ states, respectively, at the ADC(2)/cc-pVTZ level. Subsequent evaluations using DFT/PBE0-computed ϵ+ERI MolOrbImages demonstrate improved prediction accuracy (MAEs of 0.080 and 0.069 eV for $E_{S_{1}}$ and $E_{T_{1}}$ , respectively) and transferability.^43^ Of a greater importance, we designed a new class of multi-channel *ϵV* MolOrbImages that incorporates the electronic potentials (*V*) as additional channels. Separate ϵ+ERI features in ref. 42 are reconfigured into a single *ϵV* image, which enables the application of widely used VGG (Visual Geometry Group)-type^44^ CNN architectures. Quantum information associated with hole and particle states during electronic transitions is further formulated as *V* ^H^ and *V* ^P^ channels (matrix representation under the MO basis, see Section 2.1 for details), leading to *ϵV* ^H^ *V* ^P^ MolOrbImages. When connected with a revised VGG (rVGG) model, this approach achieves MAEs of <0.08 eV and <0.1 eV for excited-state energies of QM9 molecules and practical photofunctional materials (Figure 1a) with up to 560 atoms, respectively.

**Figure 1:**
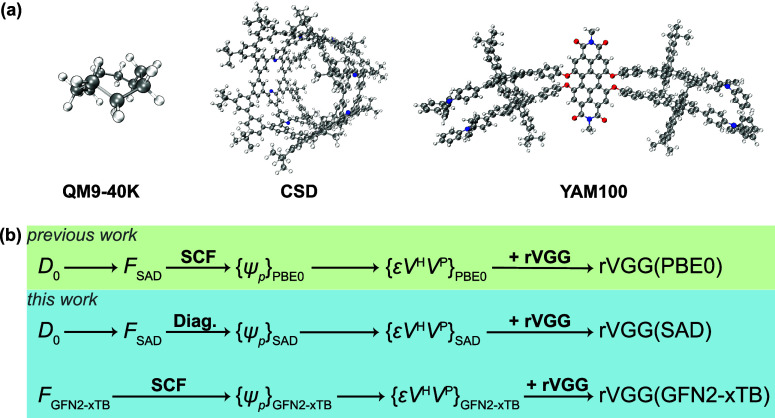
(a) Data sets and (b) rVGG models used in this work. Structure of the largest molecule (number of atoms = 27, 560, and 470 for QM9-40K, CSD, and YAM100 data sets, respectively) within each data set is presented. The rVGG­(PBE0) model is taken from ref. 43.

While our previous models can reduce the computational cost of excited-state calculations to a level comparable with that of MO calculations, fully convergent mean-field Hartree–Fock or DFT calculations remain expensive for very large molecules, such as the examples illustrated in Figure 1a. Herein, we explore low-cost schemes for generating approximate MOs, including initial guess techniques^45^ and semiempirical quantum mechanical theories.^46^ The resulting *ϵV* ^H^ *V* ^P^ MolOrbImages derived from these crude MOs are connected with the rVGG model. The predictive performance of cost-effective rVGG models is validated on small organic molecules and practical photofunctional materials. Additionally, perturbation analysis of *ϵV* ^H^ *V* ^P^ images assists in the identification of critical quantum chemical features influencing the prediction accuracy. These findings highlight the potential for rapid and accurate prediction of excited-state properties, and provide new directions for further enhancement of model performance within a cost-effective ML framework.

## 2 Theoretical and Computational Details

In this work, the occupied, unoccupied, and general MOs (ψ) are labeled by {*i*, *j*, ···}, {*a*, *b*, ···}, and {*p*, *q*, ···}, respectively. The atomic orbitals (AOs) are labeled by {λ, μ, ···}. The matrix representation of an operator *Ô* is *O*, with the matrix elements *O*_*pq*_/*O*_*λμ*_ under the MO/AO basis.

### 2.1 Cost-Effective MolOrbImage Representations

The smallest addressable unit (also referred to as pixel) of the one-electron MolOrbImage is defined as the combination of bra and ket MO states, i.e., ⟨*p*|·|*q*⟩. The height and width of the one-electron MolOrbImage are determined by a fixed even number, *N*_MO_. Since low-lying excited states mainly involve transitions among frontier orbitals, we have utilized a reduced MO space, comprising $\frac{N_{\mathrm{MO}}}{2}$ occupied and $\frac{N_{\mathrm{MO}}}{2}$ unoccupied orbitals around the Fermi level, to construct the MolOrbImage. For molecules with the number of occupied/unoccupied orbitals $<\frac{N_{\mathrm{MO}}}{2}$ , the remaining pixels of the MolOrbImage are padded with zero. In a multi-channel MolOrbImage, channels are distinguished by the nature of quantum mechanical operators used. For example, in three-channel *ϵV* ^H^ *V* ^P^ MolOrbImages, the elements in the ϵ channel correspond to one-electron ⟨*p*|·|*q*⟩ under the molecular integration with the Fock operator (*F̂*),

1 $$\epsilon_{pq}=\left\langle p\right|\mathrm{F̂}\left|p\right\rangle \delta_{pq}$$

Thus, the ϵ channel is a diagonal matrix of MO energies. The potential (*V*) channel involves an electron density [*ρ*(**r**)]-dependent term

2 $$V_{pq}=\left|\left\langle p\right|\mathrm{V̂}\left|q\right\rangle \right|$$

3 $$\mathrm{V̂}\left(r\right)=\int dr^{\prime }\rho \left(r^{\prime }\right)|r-r^{\prime }{|}^{-1}+{\mathrm{V̂}}_{\mathrm{XC}}\left(r\right)$$

Here, |**r** – **r′**|^–1^ is the electron–electron Coulomb repulsion operator, and *V̂*_XC_(**r**) denotes the exchange–correlation potential, which is the functional derivative of the exchange–correlation energy (*E*_XC_) with respect to the electron density^47^

4 $${\mathrm{V̂}}_{\mathrm{XC}}\left(r\right)=\frac{\delta E_{\mathrm{XC}}\left[\rho \left(r\right)\right]}{\delta \rho \left(r\right)}$$

To compute *V* channels for hole (*V* ^H^) and particle (*V* ^P^) states, normalized hole and particle density matrices are defined as

5 $$D_{\lambda \mu }^{H}=\frac{\underset{i}{\sum }2C_{\lambda i}^{*}C_{\mu i}\cdot \exp\left[-\beta \left(\epsilon_{\mathrm{HOMO}}-\epsilon_{i}\right)\right]}{\underset{i}{\sum }\exp\left[-\beta \left(\epsilon_{\mathrm{HOMO}}-\epsilon_{i}\right)\right]}$$

6 $$D_{\lambda \mu }^{P}=\frac{\underset{a}{\sum }2C_{\lambda a}^{*}C_{\mu a}\cdot \exp\left[\beta \left(\epsilon_{\mathrm{LUMO}}-\epsilon_{a}\right)\right]}{\underset{a}{\sum }\exp\left[\beta \left(\epsilon_{\mathrm{LUMO}}-\epsilon_{a}\right)\right]}$$

In these expressions, *C* represents the MO coefficient, and ϵ_HOMO_ and ϵ_LUMO_ are the energies of the highest occupied MO (HOMO) and lowest unoccupied MO (LUMO), respectively. The factor $\beta =\frac{1}{k_{B}T}$ , where *k*_B_ is the Boltzmann constant, serves as a hyperparameter that modulates the contribution of occupied and unoccupied orbitals to the hole and particle densities, based on their energy difference relative to HOMO and LUMO. In our previous work,^43^ the ϵ, *V* ^H^, and *V* ^P^ channels were evaluated using converged PBE0 orbitals, resulting in PBE0-based *ϵV* ^H^ *V* ^P^ images. To facilitate the learning of molecular excited states, we adopted the rVGG model in conjunction with *ϵV* ^H^ *V* ^P^ descriptors. We refer to this combined approach as the rVGG­(PBE0) model (Figure 1b).

However, the convergence of PBE0 orbitals involves numerous self-consistent field (SCF) cycles, which can be computationally demanding for molecules with >100 atoms. This challenge can be addressed from several perspectives. For instance, using minimal basis sets such as STO-3G can reduce the computational cost, though SCF procedures remain computationally formidable for very large systems. To limit the number of SCF cycles, it is advantageous to generate high-quality initial guess orbitals from a guess Hamiltonian or density matrix. Various approaches for initial guesses have been extensively studied, including the one-electron guess, the generalized Wolfsberg–Helmholz approximation,^48^ the extended Hückel methods,^49^ and the superposition of atomic densities (SAD).^50^ A comprehensive review of initial guess strategies can be found in ref. 45. In this work, we focus on the SAD method, which constructs a nonidempotent initial guess density matrix (*D*_0_) from converged atomic density matrices at each nucleus. To mitigate issues arising from nonidempotency, we have implemented a procedure similar to that reported in ref. 50 where a spin-restricted PBE0 Fock matrix (*F*_SAD_) is built using *D*_0_. Diagonalization of *F*_SAD_ yields a set of SAD/PBE0 orbitals. These orbitals, which can be used to form an idempotent density matrix, are then applied to generate SAD-based *ϵV* ^H^ *V* ^P^ images. The integration of this approach with the rVGG model results in the rVGG­(SAD) model. In contrast to the rVGG­(PBE0) model, which requires multiple SCF cycles, the rVGG­(SAD) model necessitates only a single diagonalization step (Figure 1b). However, the rVGG­(SAD) model now also involves additional effort to map crude S_0_ state information to excited-state properties, whereas the S_0_ information in the rVGG­(PBE0) model is inherently more accurate.

Besides the initial guess techniques, another class of computationally efficient methods for generating approximate orbitals is semiempirical quantum mechanical theories.^46^ Among these, we are particularly interested in the recently proposed GFN2 (geometries, frequencies, and noncovalent interactions)-xTB method, as it can reasonably describe the S_0_ state of organic, organometallic, and biochemical systems with up to 1,000 atoms. Unlike the SAD approach, the GFN2-xTB method requires SCF convergence to generate orbitals, while the calculation can be completed within seconds. A detailed introduction of the method is provided in ref. 51. In our approach, the ϵ channel uses energies of GFN2-xTB-derived orbitals. We have computed *D* ^H^ and *D* ^P^ at the GFN2-xTB level based on eqs 5 and 6, and evaluated *V* ^H^ and *V* ^P^ within the PBE0 framework. These calculations yield GFN2-xTB-based *ϵV* ^H^ *V* ^P^ images, which are connected with the rVGG­(GFN2-xTB) model (Figure 1b).

### 2.2 Data Sets and Machine Learning Model

In this study, three data sets have been utilized (Figure 1a). The QM9-40K data set and its extrapolation set (QM9-ES) are obtained from ref. 42. The CSD data set,^52^ which includes 48,075 organic semiconductor (OSC) materials with a stable S_0_ state ( $E_{T_{1}}$ > 0 eV) sourced from the Cambridge Structural Database,^53^ is taken from ref. 43. Additionally, we have collected a series of 100 organic photofunctional materials (YAM100) reported by Yam and co-workers (Table S1). For the YAM100 data set, the first 30 compounds are adopted from ref. 43 and Cartesian coordinates of the remaining 70 compounds are provided in the Supporting Information.

The rVGG model features a convolutional block designed to extract important features from *ϵV* ^H^ *V* ^P^ images, followed by a fully connected block for the prediction of excited-state energies. The output layer consists of six nodes, corresponding to the energies of S_*n*_ and T_*n*_ (*n* = 1–3) states. All layers are activated with a ReLU (rectified linear unit) function, and additional batch normalization is applied to the fully connected layers. The detailed architecture of the rVGG model is described in ref. 43. Implementation has been conducted using the PyTorch^54^ and PyTorch Lightning^55^ packages.

### 2.3 Computational Details

For molecules in the YAM100 data set, the S_0_ structures are optimized at the PBE0-D3BJ/6-31G­(d,p) level using the Gaussian 16 (revision C.01) package.^56^ Excited-state properties at the TDA/PBE0/def2-SVP and ZINDO/S^57^ (Zerner’s intermediate neglect of differential overlap with singles) levels are computed with the ORCA (release 5.0.3) package.^58^ For xTB-sTDA excited-state data, xtb4stda (version 1.0) program^59^ is used to prepare the wave function output, and stda (version 1.6.2) program^60^ is used to compute singlet and triplet excited-state energies with an energy threshold of 10 eV. Initial guess orbitals (SAD/PBE0/cc-pVTZ and SAD/PBE0/STO-3G for QM9-40K, and SAD/PBE0/def2-SVP for CSD and YAM100) are evaluated using the PySCF (version 2.1.1) package.^61^ GFN2-xTB orbitals are obtained via the standalone xtb (version 6.5.0) code,^62^ and then saved in the MOLDEN files, which are further standardized by the Multiwfn (version 3.8dev) package.^63^ The *V* ^H^ and *V* ^P^ terms are computed using the get_veff­(ks, dm) function within the pyscf.dft module by setting dm = *D* ^H^ and *D* ^P^, respectively. Here, ks is a Kohn–Sham DFT object with the PBE0 approximation.

To ensure a fair comparison of prediction accuracy with the rVGG­(PBE0) model, consistent training–validation–test splitting strategies as described in refs. 42 and 43 have been adopted for the QM9-40K and CSD data sets, respectively, for training the rVGG­(SAD) and rVGG­(GFN2-xTB) models. Specifically, the QM9-40K data set contains 32,000 training molecules, 3,200 validation molecules, and 4,800 test molecules, while the CSD data set contains 40,000 training molecules, 4,000 validation molecules, and 4,075 test molecules. The training loss is evaluated using RMSE, and the AdamW^64^ optimizer is used for up to 2,000 epochs. The batch size is set to 128. An initial learning rate (LR) of 0.001 is adopted, and the LR would be reduced by 20% (until it reaches 1 × 10^–6^) if the validation loss does not improve after 10 consecutive epochs. To enhance the rVGG­(GFN2-xTB) model for the CSD data set, we first train the rVGG­(PBE0) model with identical hyperparameters (β and *N*_MO_) using the *ϵV* ^H^ *V* ^P^ images derived from converged PBE0/def2-SVP orbitals. The optimized rVGG­(PBE0) parameters are adopted from ref. 43. Subsequently, we retrain the rVGG­(PBE0) model with GFN2-xTB computed *ϵV* ^H^ *V* ^P^ images, allowing all rVGG parameters to be fine-tuned. The initial LR is reduced to 1 × 10^–4^ to facilitate mild optimization of the model parameters, leading to the rVGG­(GFN2-xTB) model via transfer learning. All other settings remain consistent with those used in the direct training approach. The overall performance of the rVGG model is assessed by examining the *L*_1_ deviation in predicting excitation energies for the S_0_ → S_*n*_ and S_0_ → T_*n*_ (*n* = 1–3) transitions, relative to the theoretically computed reference

7 $$L_{1}=\frac{1}{6N_{\mathrm{test}}}\overset{3}{\underset{n}{\sum }}\overset{N_{\mathrm{test}}}{\underset{i}{\sum }}\left|E_{S_{n}}^{\mathrm{rVGG}}\left(i\right)-E_{S_{n}}^{\mathrm{ref}}\left(i\right)\right|+\left|E_{T_{n}}^{\mathrm{rVGG}}\left(i\right)-E_{T_{n}}^{\mathrm{ref}}\left(i\right)\right|$$

where *N*_test_ is the number of molecules in the test set.

## 3 Results and Discussion

Unless otherwise specified, the reported results in this section are out-of-sample predictions; that is, molecules in both the training and validation sets are excluded from the evaluation.

### 3.1 Benchmark Study on Small Organic Molecules

Using the QM9-40K data set, a systematic grid search has been conducted over hyperparameters β and *N*_MO_ for the *ϵV* ^H^ *V* ^P^+rVGG models, utilizing MOs derived from SAD/PBE0/cc-pVTZ and GFN2-xTB calculations. Optimal β and *N*_MO_ values are determined by analyzing the *L*_1_ deviation as defined in eq 7, with the reference computed at the ADC(2)/cc-pVTZ level. The number of basis functions (*N*_BF_) associated with the largest molecule in QM9-40K at the GFN2-xTB level (54, Table S2) is substantially smaller than that at the SAD/PBE0/cc-pVTZ level (522), as a minimal spherical valence basis set is adopted in GFN2-xTB.^51^ Consequently, rVGG­(GFN2-xTB) model involves a maximal *N*_MO_ value of 60. For small organic molecules, *N*_MO_ significantly influences model performance, with the *L*_1_ deviation of <0.1 eV achieved at large *N*_MO_ values of 80 and 60 for rVGG­(SAD) and rVGG­(GFN2-xTB) models, respectively, regardless of the β value (Figure S1 and Table S3). The rVGG­(SAD) model based on SAD/PBE0/cc-pVTZ guess orbitals achieves optimal performance at β = 10 Hartree^–1^ and *N*_MO_ = 80, yielding a minimal *L*_1_ deviation of 0.088 eV. It is observed that the *L*_1_ deviations exhibit a strong correlation with the corresponding *L*_2_ deviations across all β and *N*_MO_ pairs (Figure S2). The model that minimizes the *L*_1_ deviation also achieves the minimal *L*_2_ deviation. The minimal *L*_1_ deviation from the rVGG­(SAD) model is slightly larger than that from the optimal rVGG­(PBE0) model (0.062 eV).^43^ Given that SAD guess orbitals are generated after only a single diagonalization step, such moderate deterioration in prediction accuracy is acceptable, especially considering the significant reduction in computational cost. For rVGG­(GFN2-xTB) models, a minimal *L*_1_ deviation of 0.093 eV has been attained at β = 10 Hartree^–1^ and *N*_MO_ = 60.

**Figure 2:**
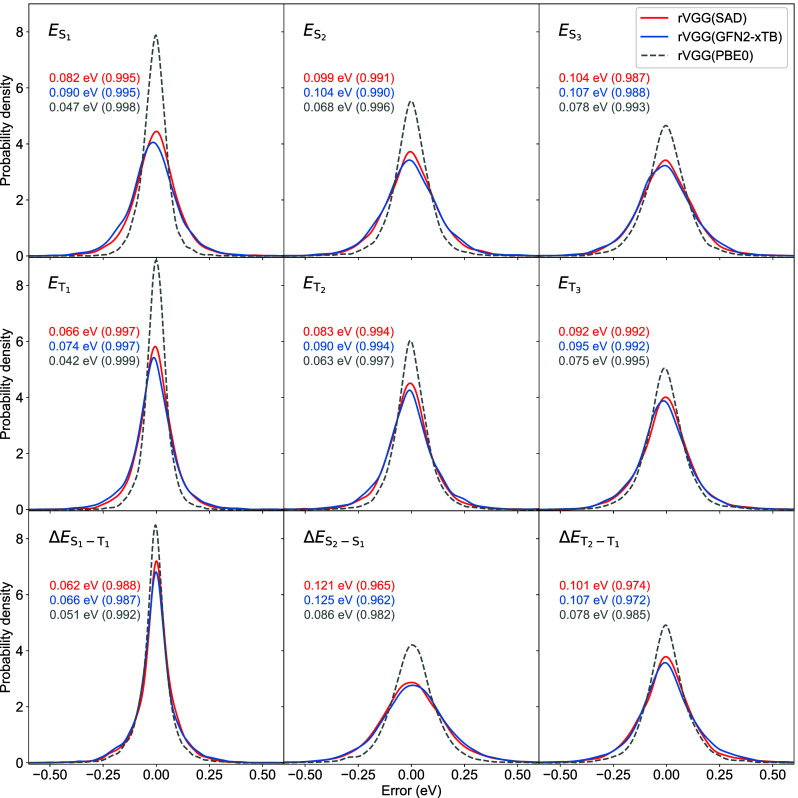
Distributions of errors in predicting excited-state properties computed at the ADC(2)/cc-pVTZ level. Results (gray dashed lines) from the optimal *ϵV* ^H^ *V* ^P^+rVGG model based on the converged PBE0/cc-pVTZ MOs are taken from ref. 43. MAE and *r* (in parentheses) values are provided. All models are trained within the QM9-40K data set with *N*_train_ = 32,000.

Focusing on the optimal models, we investigate the influence of MO quality on the accuracy of rVGG predictions for individual excited-state properties (Figure 2). The error distributions for both excitation energies and excited-state energy gaps exhibit Gaussian-like profiles centered near 0 eV (Table S4) in the middle region, as revealed from the linearity in the corresponding quantile–quantile (Q–Q) plots (Figure S3). However, curvatures at both positive and negative ends of the Q–Q plots reveal heavier-than-normal tails, indicating occasional large deviations at the extremes. These results imply that all three rVGG models demonstrate minimal bias and systematic errors, with extreme outliers at both positive and negative directions. Across nearly all models and excited-state energies, outliers with large negative errors occur more frequently than those with positive errors (Table S5). For example, at a threshold of *t* = 0.1 eV, the directional exceedance probabilities *P*(error > *t*) and *P*(error < –*t*) for errors associated with $E_{S_{1}}$ predicted by the rVGG­(GFN2-xTB) model are 0.135 and 0.195, respectively, indicating that negative exceedances are approximately 45% more frequent. Overall, the magnitude of the standard deviation serves as an indicator of model performance (Table S4). The optimal rVGG­(SAD) model with SAD/PBE0/cc-pVTZ-computed *ϵV* ^H^ *V* ^P^ images marginally outperform the rVGG­(GFN2-xTB) model, as evidenced by slightly lower MAEs and higher *r* values for the excited-state properties (Table S6) under investigation. Comparison with the optimal rVGG­(PBE0) model indicates that the application of semiempirical GFN2-xTB or SAD guess orbitals results in a more pronounced performance degradation to the lowest-lying S_1_ and T_1_ excited states, with standard deviations of errors between rVGG­(GFN2-xTB) and rVGG­(PBE0) differing by 0.051 and 0.040 eV, respectively (Table S4). This discrepancy diminishes as *n* increases from 1 to 3, ultimately reaching 0.036 and 0.025 eV for $E_{S_{3}}$ and $E_{T_{3}}$ , respectively. For excited-state energy gaps, the optimal rVGG model suffers from minor performance degradation in predicting $\Delta E_{S_{1}-T_{1}}$ upon switching from converged PBE0 orbitals to semiempirical GFN2-xTB or SAD guess orbitals, as reflected by comparable MAE values among SAD (0.062 eV, Table S6), GFN2-xTB (0.066 eV), and PBE0 (0.051 eV). Notably, larger reductions in model performance are observed for $\Delta E_{S_{2}-S_{1}}$ and $\Delta E_{T_{2}-T_{1}}$ . For the rVGG­(SAD) model, performance remains largely unchanged upon switching from SAD/PBE0/cc-pVTZ-computed *ϵV* ^H^ *V* ^P^ images to SAD/PBE0/STO-3G counterparts. The deviations in MAE and *r* values are within 0.006 eV and 0.003, respectively, across all excited-state properties (Table S7).

With a focus on $E_{S_{1}}$ , we further identify extreme outliers as suggested from the Q–Q plots for both the rVGG­(SAD) and rVGG­(GFN2-xTB) models. For rVGG­(SAD), all outliers with underestimated $E_{S_{1}}$ correspond to saturated molecules containing trifluoromethyl or epoxy groups (Figure S4a) while overestimated outliers are primarily unsaturated molecules (Figure S4b). Substantial overestimations (>0.8 eV) are observed for conjugated systems with diffuse π-electrons. Our finding is consistent with the analysis of DFT outliers relative to the CC2 calculated $E_{S_{1}}$ values.^29^ Conversely, the behavior of the rVGG­(GFN2-xTB) model is less predictable. Among its underestimated outliers, in addition to saturated molecules with trifluoromethyl or epoxy groups, it performs poorly on unsaturated molecules featuring C≡C or C≡N triple bonds (Figure S5a). Regarding overestimated outliers, most are unsaturated molecules with π-conjugation, though it can occasionally overestimate $E_{S_{1}}$ by >0.6 eV for saturated molecules, such as molecule **041044** in the QM9-40K data set (Figure S5b).

The transferability of the *ϵV* ^H^ *V* ^P^+rVGG models has been quantified through performance assessments on the QM9-ES data set.^42^ With varying values of *N*_MO_, the same β value as in Figure 2 is adopted. Consistent with our previous observations,^43^ the *L*_1_ deviations generally increase with larger *N*_MO_ values (Table S8), indicating a diminished extrapolation capability of the rVGG model in the presence of large *ϵV* ^H^ *V* ^P^ images. To facilitate a more detailed analysis, the predictions from the model with the lowest *L*_1_ deviation, which correspond to *N*_MO_ values of 20, 20, and 40 for SAD, GFN2-xTB, and PBE0, respectively, are extracted (Table S9). Although models based on SAD and GFN2-xTB-derived orbitals exhibit comparable accuracy for QM9 molecules, the rVGG­(GFN2-xTB) model demonstrates markedly superior performance on out-of-sample molecules that significantly differ from molecules in the QM9-40K data set, as evidenced by MAE < 0.34 eV and *r* > 0.92 across all low-lying excited-state energies. Notably, the rVGG­(GFN2-xTB) model achieves promising accuracy for $E_{T_{1}}$ (MAE = 0.255 eV and *r* = 0.965, Figure 3), and the decline in performance relative to the rVGG­(PBE0) model (MAE = 0.172 eV, Table S9) is within 0.1 eV. In sharp contrast, the rVGG­(SAD) model exhibits MAE > 0.49 eV and *r* < 0.92, indicating that the model transferability tends to decrease when applying SAD guess orbitals that lack SCF convergence. In practical applications, it is recommended to use the rVGG­(GFN2-xTB) model with smaller *N*_MO_ values of 20 or 40 for out-of-sample molecules.

**Figure 3:**
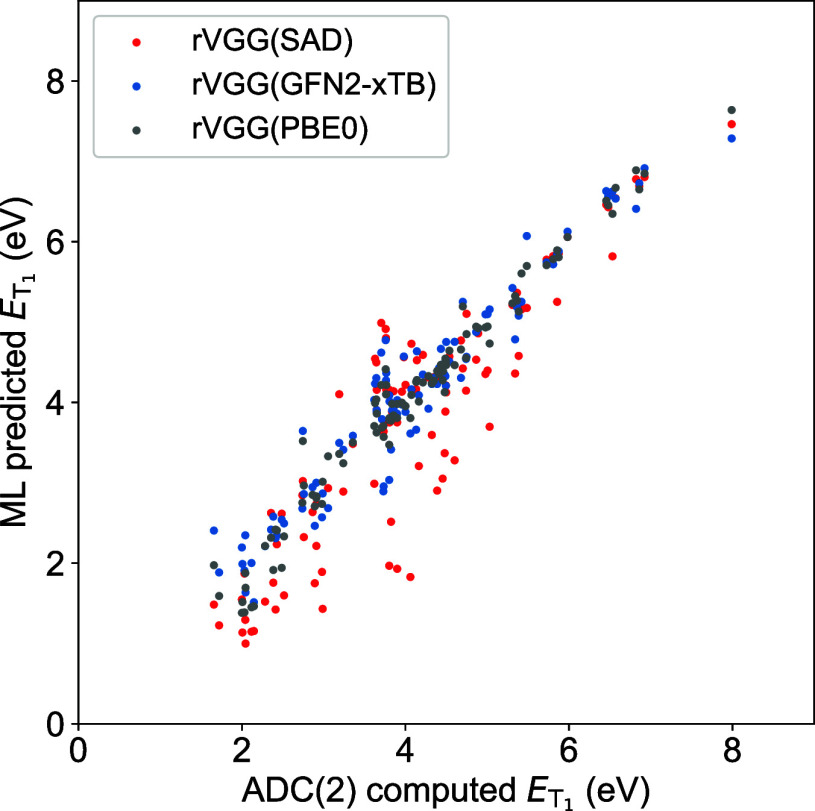
Correlation between the rVGG prediction and ADC(2)-computed reference for $E_{T_{1}}$ of molecules in the QM9-ES data set.

### 3.2 Application to Practical Photofunctional Materials

A similar hyperparameter tuning procedure aimed at optimizing β and *N*_MO_ has been conducted on the practical CSD data set (Figures 4, S6 and Table S10). The optimal values (β = 100 Hartree^–1^ and *N*_MO_ = 100) are consistently identified for both rVGG­(SAD) and rVGG­(GFN2-xTB) models (Figure S7). While the models achieve comparable accuracy on the QM9-40K data set, their performance diverges on the CSD data set. Specifically, optimal rVGG models based on SAD (0.186 eV) and GFN2-xTB (0.130 eV) orbitals exhibit distinct differences in the overall *L*_1_ deviation. Using the rVGG­(GFN2-xTB) model, the MAEs for the excitation energies of the lowest-lying S_1_ and T_1_ states are approximately 0.12 eV (Table S11), while slightly larger errors (0.133–0.137 eV) are observed for energetically higher-lying states ( $E_{S_{n}}$ and $E_{T_{n}}$ , *n* = 2 and 3). The performance degradation compared to the rVGG­(PBE0) model is about 0.06 eV in MAEs for excitation energies (Table S11). Additionally, a strong correlation (*r* > 0.9) is observed between the rVGG­(GFN2-xTB) prediction and the TDA/PBE0 computed reference. In contrast, the rVGG model in connection with SAD orbitals demonstrates less promising predictive capability (MAE > 0.17 eV and *r* < 0.87) for excitation energies of practical photofunctional materials. This limitation restricts its application for the prescreening of photofunctional candidates in the vast chemical compound space.

**Figure 4:**
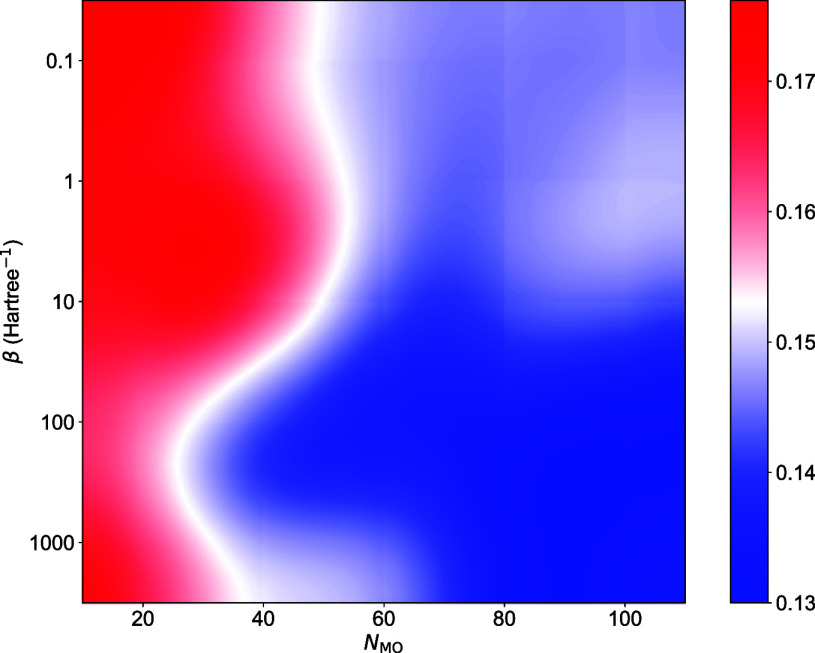
*L*_1_ deviations (eV) of the rVGG­(GFN2-xTB) prediction as a function of β and *N*_MO_ for the CSD data set.

The performance of the rVGG­(SAD) model in predicting excited-state energy gaps is only marginally satisfactory, particularly for $\Delta E_{S_{2}-S_{1}}$ (*r* = 0.650) and $\Delta E_{T_{2}-T_{1}}$ (*r* = 0.774). The incorporation of GFN2-xTB computed *ϵV* ^H^ *V* ^P^ images enhances the correlation coefficients to 0.817 and 0.849 for the singlet–singlet and triplet–triplet gaps, respectively. A strong correlation (*r* = 0.909) is achieved for $\Delta E_{S_{1}-T_{1}}$ , which is likely due to smaller prediction errors associated with $E_{S_{1}}$ and $E_{T_{1}}$ . Notably, the utilization of a combined RMSE loss function on both excited-state energies (loss weight = 1) and gaps (loss weight = 20) results in slight reductions in MAEs for $\Delta E_{S_{1}-T_{1}}$ , $\Delta E_{S_{2}-S_{1}}$ and $\Delta E_{T_{2}-T_{1}}$ by about 0.01 eV, with *r* values improving to 0.916, 0.831, and 0.872, respectively (Figure S8). The prediction accuracy for excited-state energies experiences only minor deterioration. However, further development of the *ϵV* ^H^ *V* ^P^+rVGG architecture with computationally efficient MOs is necessary to achieve higher correlation levels (*r* > 0.92) comparable to those obtained with the optimal rVGG­(PBE0) model across all excited-state energy gaps (Table S11).

We have further applied the optimal rVGG­(GFN2-xTB) model (β = 100 Hartree^–1^ and *N*_MO_ = 100) to the YAM100 data set (Tables S12 and S13). The YAM100 test set includes photochromic diarylethenes, heterocyclic spiro derivatives, circularly polarized luminescence-active [*n*]­cyclo-paraphenylene-pillar­[5]­arene bismacrocycles, boron­(III)-based TADF emitters, and BODIPY (4,4-difluoro-4-bora-3a,4a-diaza-*s*-indacene) derivatives, extending beyond the OSC materials in the CSD data set. For the low-lying S_1_–S_3_ states (Figures 5a, S9a and S9b), the rVGG model combined with semiempirical GFN2-xTB *ϵV* ^H^ *V* ^P^ yields excitation energy predictions with errors <0.15 eV for excitation energies, comparable to those for the CSD data set (Table S11). The performance deviation from the rVGG­(PBE0) model using converged PBE0/def-SVP orbitals, as measured by MAE and *r*, remains within 0.075 eV and 0.04, respectively. Focusing on the best-predicted excited state $\left(E_{S_{1}}\right)$ , outliers (Figure 6 and Table S14) with rVGG prediction deviations exceeding 0.3 eV from TDA/PBE0 reference values are analyzed. With *N*_MO_ = 100, the rVGG model contains approximately 260 million trainable parameters, posing a significant risk of overfitting (Figure S10). This overfitting concern is especially relevant for molecules with small number of occupied orbitals (*N*_OCC_), such as compounds **9**,^65^ **43** ^6^ and **77**.^66^ Switching to the rVGG­(GFN2-xTB) model with *N*_MO_ = 60 (122 million trainable parameters) largely mitigates overfitting for small molecules, while deviations from reference values are still >0.3 eV for some compounds with large *N*_OCC_ like **29** ^67^ and **90**.^68^ Regarding the application of the optimal rVGG­(GFN2-xTB) model to predict $E_{T_{n}}$ (*n* = 1–3, Figures 5b, S9c and S9d), the MAEs for the YAM100 data set are clearly higher than those for the CSD data set, suggesting a reduced transferability of the rVGG­(GFN2-xTB) model for excitation energies of triplet states.

**Figure 5:**
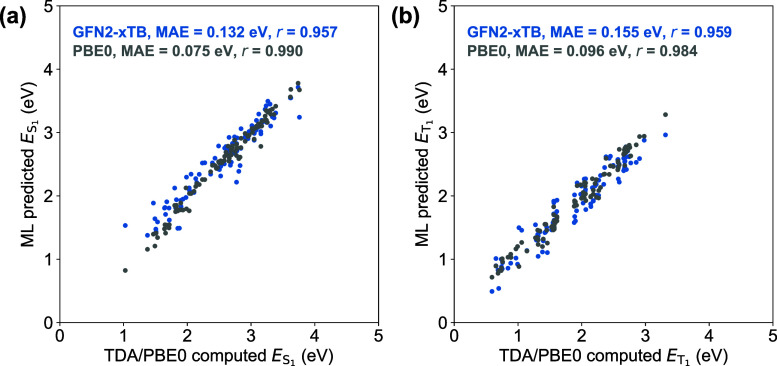
Correlation between the rVGG prediction and TDA/PBE0-computed reference for (a) $E_{S_{1}}$ and (b) $E_{T_{1}}$ of 100 organic photofunctional materials in the YAM100 data set.

**Figure 6:**
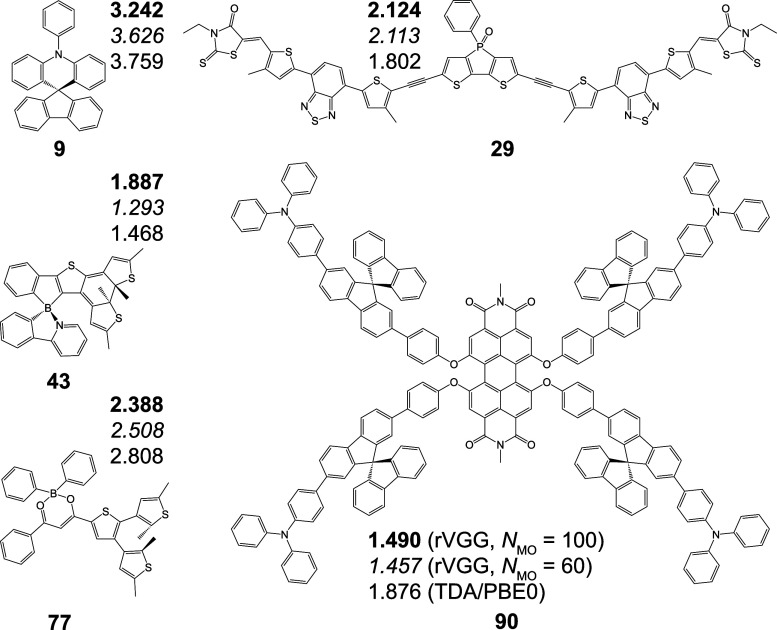
Selected molecular structures of outliers for the optimal rVGG­(GFN2-xTB) model in predicting $E_{S_{1}}$ . Predictions from rVGG­(GFN2-xTB) models with *N*_MO_ = 100 (bold) and 60 (italic) are compared with the TDA/PBE0-computed values (regular).

### 3.3 Perturbation Analysis on MolOrbImage Representations

To clarify the contribution component of the *ϵV* ^H^ *V* ^P^ that significantly influences the prediction accuracy of the rVGG model within the CSD data set, we have evaluated the percentage change in the *L*_1_ deviation (%Δ*L*_1_) of the test set resulting from perturbations [Δ­(*ϵV* ^H^ *V* ^P^)] applied to the MolOrbImage representations

8 $$\%\Delta L_{1}=\frac{L_{1}\left[\epsilon V^{H}V^{P}+\Delta \left(\epsilon V^{H}V^{P}\right)\right]-L_{1}\left(\epsilon V^{H}V^{P}\right)}{L_{1}\left(\epsilon V^{H}V^{P}\right)}\times 100\%$$

Specifically, we have first conducted channel-wise perturbations, where Δ­(*ϵV* ^H^ *V* ^P^) = *δ* · *ϵ* (Figure 7a) or *δ* · *V* ^H^ (Figure 7b) or *δ* · *V* ^P^ (Figure 7c) while keeping the other two channels unchanged. In this approach, all elements within the perturbed channel are scaled by a factor of 1 + *δ* relative to their original magnitudes. Our results demonstrate that for both the rVGG­(SAD) and rVGG­(GFN2-xTB) models, perturbations to the MO energies induce a substantial increase in the *L*_1_ deviation. When all MO energies are attenuated simultaneously by 15%, the initial *L*_1_ deviation of 0.134 eV associated with the rVGG­(GFN2-xTB) model (β = 100 Hartree^–1^ and *N*_MO_ = 60) increases by 123.1% to 0.298 eV. Comparison of the percentage changes across the three channels indicates that ϵ is the most influential component of the MolOrbImage representations. Within the CSD test set, the rVGG­(GFN2-xTB) model exhibits greater sensitivity to perturbations in ϵ compared to the rVGG­(SAD) model. This may be attributed to the fact that MO energies derived from self-consistent GFN2-xTB calculations are less random (Figures S11a and S11b), which makes them more susceptible to noise-like perturbations. Conversely, energies from SAD guess orbitals are inherently more stochastic and thus more tolerant to such perturbations. Interestingly, the sensitivity of both models to ϵ-perturbations is nearly the same within the QM9-40K data set (Figure S12a), which is consistent with the similar levels of randomness observed in MO energies derived from SAD and GFN2-xTB calculations (Figures S11c and S11d). This observation correlates with the finding that, though the performances of rVGG­(SAD) and rVGG­(GFN2-xTB) are comparable on the QM9-40K data set, the divergence in prediction accuracy becomes apparent within the CSD data set. This underscores the importance of validating ML models with practical systems, as promising performance on benchmark molecules does not necessarily guarantee reliable results for practically useful materials. Further element-wise perturbation analysis of MO energies [Δ­(*ϵV* ^H^ *V* ^P^) = 0.05 · *ϵ_p_*] indicates that the energies of frontier orbitals near the Fermi level, particularly the HOMO and LUMO, are more important features for the rVGG model (Figure 7d).

**Figure 7:**
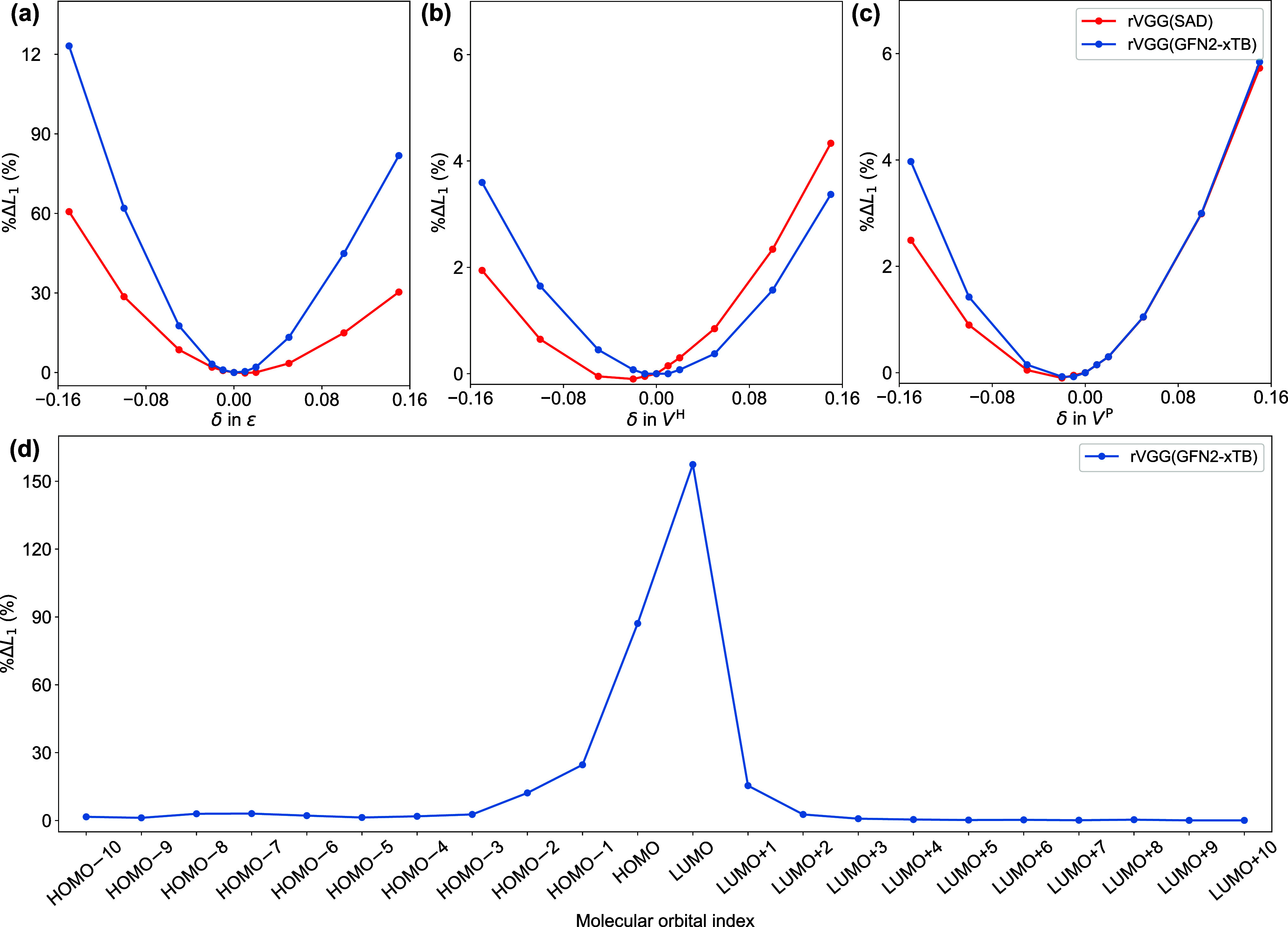
%Δ*L*_1_ resulting from (a) ϵ, (b) *V* ^H^ and (c) *V* ^P^ channel-wise perturbations and (d) ϵ_*p*_ element-wise perturbations applied to *ϵV* ^H^ *V* ^P^ for the CSD data set.

Results from perturbation analysis highlight the significance of MO energies for the rVGG model. It is reasonable to hypothesize that the performance of the rVGG­(GFN2-xTB) could be further improved by incorporating information from more accurate PBE0-converged orbitals. To this end, we have applied a transfer learning approach, initializing the rVGG­(GFN2-xTB) model with weights derived from the rVGG­(PBE0) model. Unlike directly integrating PBE0 orbital information into the GFN2-xTB computed *ϵV* ^H^ *V* ^P^ images, this transfer learning strategy only requires computationally expensive DFT calculations for both training and validation sets. At the inference stage, computationally inexpensive GFN2-xTB calculations are sufficient for the generation of input features. Applying transfer learning to the rVGG­(GFN2-xTB) models with *N*_MO_ = 60, 80, and 100 on the CSD data set indeed leads to reductions of the *L*_1_ deviation from 0.134, 0.133, and 0.130 eV to 0.123, 0.120, and 0.119 eV, respectively. More importantly, for the YAM100 data set, the previously observed overfitting issue in the direct learning approach is partially alleviated via transfer learning (Figure S10). For extremely large compounds **90** (1.490 eV, Table S14) and **91** ^68^ (1.488 eV), the severely underestimated $E_{S_{1}}$ values predicted at *N*_MO_ = 100 have been corrected to 1.936 and 1.837 eV, respectively. The overall MAE in predicting $E_{S_{1}}$ for the YAM100 data set decreases from 0.132 to 0.114 eV (Figure S10). Building on our preliminary findings, encoding DFT/PBE0-computed features into the semiempirical *ϵV* ^H^ *V* ^P^ ML architecture to approach the accuracy of the rVGG­(PBE0) model merits further investigation.

### 3.4 Comparison with Semi-Empirical Excited-State Methods

The accuracy of our rVGG­(GFN2-xTB) model, developed via transfer learning, has been evaluated through a comparative study with semiempirical excited-state methods, including ZINDO/S and xTB-sTDA. It should be noted that the ZINDO/S model is not implemented for compounds containing germanium or selenium, including **16**, **31**–**34**, and **41** in the YAM100 data set. Targeting at TDA/PBE0-computed singlet excited-state energies (Figures 8a and S13), the rVGG formalism achieves superior performance (MAE < 0.14 eV) compared to ZINDO/S (MAE > 0.29 eV) and xTB-sTDA (MAE > 0.32 eV). The predicted $E_{S_{1}}-E_{S_{3}}$ energies from rVGG exhibit strong correlation (*r* > 0.95) with the computational reference, whereas *r* coefficients for ZINDO/S (<0.82) and xTB-sTDA (<0.87) predictions are comparatively lower. Additionally, ZINDO/S fails to produce reliable triplet excited-state energies, with MAE values reaching approximately 1 eV (Figures 8b and S13). The xTB-sTDA approach demonstrates higher reliability (*r* = 0.948) for $E_{T_{1}}$ . It yields MAE of 0.283 eV, which is evidently larger than that of our model (0.148 eV). As an illustrative application, we have applied our rVGG­(GFN2-xTB) model, alongside ZINDO/S and xTB-sTDA methods, to estimate the emission energies of boron­(III)-based TADF emitters (compounds **4**, **21**, **22**, and **93**–**100** in the YAM100 data set) in toluene solution (Figure 8c and Table S15).^9,10^ For compounds **4** (λ_em_ = 537 nm, 2.309 eV) and **94** (λ_em_ = 457 nm, 2.713 eV) featuring distinct emission colors, ZINDO/S (3.129 and 3.150 eV) and xTB-sTDA (3.330 and 3.397 eV) yield nearly identical $E_{S_{1}}$ energies, in line with the poor correlation level of *r* < 0.5 between theoretical results and experimental values. In sharp contrast, the rVGG model provides a reasonable description of the emission energies based on the predicted $E_{S_{1}}$ energies. The strikingly high correlation (*r* = 0.927) enables the application of a linear calibration scheme using merely two experimental data points. By correlating experimental emission energies with rVGG derived $E_{S_{1}}$ values for **4** and **21**, a linear relationship is established to estimate the emission energies of the remaining compounds, achieving a rather small MAE of 0.052 eV (Figure 8d). However, such calibration scheme cannot be reliably applied to semiempirical excited-state methods due to their limited accuracy in practical applications.

**Figure 8:**
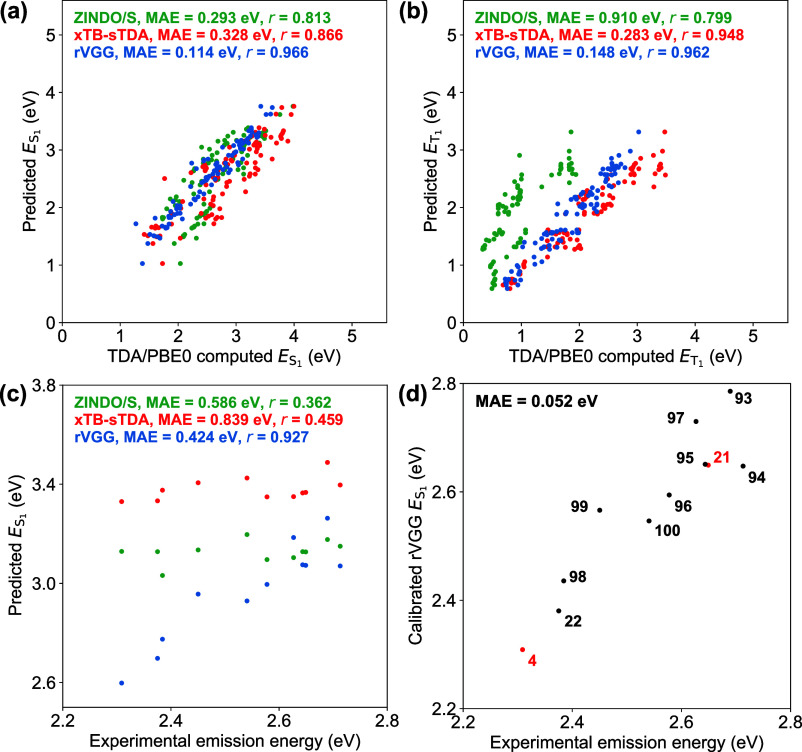
Performance of the rVGG­(GFN2-xTB) model versus semiempirical excited-state methods. Theoretical predictions from ZINDO/S, xTB-sTDA, and rVGG approaches are compared with TDA/PBE0-computed reference for (a) $E_{S_{1}}$ and (b) $E_{T_{1}}$ of 100 organic photofunctional materials in the YAM100 data set. (c) ZINDO/S, xTB-sTDA, and rVGG methods are applied to predict emission energies of boron­(III)-based TADF emitters in toluene solution. (d) rVGG predicted $E_{S_{1}}$ values are calibrated with experimental emission energies. Transfer learning technique has been applied to train the rVGG­(GFN2-xTB) model. Here, β = 100 Hartree^–1^ and *N*_MO_ = 100.

Finally, we have conducted time benchmarks to compare the computational costs associated with SAD, GFN2-xTB, and fully convergent PBE0 approaches for generating *ϵV* ^H^ *V* ^P^ MolOrbImages (Tables 1 and S16), alongside a cross-comparison with semiempirical excited-state methods. Although the molecules in the YAM100 and QM9-ES data sets differ significantly in structural complexity (Figure S14), both SAD and GFN2-xTB exhibit substantial reductions in wall time, relative to the cost of computing converged PBE0 orbitals. Notably, GFN2-xTB-derived orbitals can be obtained with marginal computational time, representing a speed-up of over 1,000-fold for YAM100 and 200-fold for QM9-ES data sets compared to PBE0 calculations. The generation of *ϵV* ^H^ *V* ^P^ requires the computation of the effective potential term as defined in eq 2, which depends on *N*_BF_. This step becomes a computational bottleneck in our rVGG­(SAD) and rVGG­(GFN2-xTB) approaches as the PBE0 hybrid functional is applied. To address this issue, ongoing efforts will be focused on implementing acceleration techniques such as density-fitting and chain-of-spheres algorithms^69^ to speed up the formation of Coulomb and exchange–correlation parts within the effective potential calculation. Currently, the wall time for the rVGG­(GFN2-xTB) model is comparable to that of xTB-sTDA method. In terms of accuracy, our results indicate that the rVGG­(GFN2-xTB) approach consistently outperforms xTB-sTDA for both the YAM100 (Figures 8 and S13) and QM9-ES (Figure S15) data sets, while ZINDO/S is unreliable for practical applications.

**Table 1:** Wall Times (s) Required to Generate the Orbitals (*ψ*) and *ϵV* ^H^ *V* ^P^ MolOrbImages and to Compute the Excited-State Energies Using Semi-Empirical Methods for the YAM100 and QM9-ES Data Sets^a^

	SAD^b^		GFN2-xTB		PBE0^b^			
	ψ	$\epsilon V^{H}V^{P}$	ψ	$\epsilon V^{H}V^{P}$	ψ	$$\epsilon V^{H}V^{P}$$	ZINDO/S	xTB-sTDA
YAM100	4,988	17,528	139	4,800	145,032	17,007	722	4,527
QM9-ES	327	1,349	34	229	7,460	1,381	246	135

a The values are averaged over four simulations.

b Def2-SVP and cc-pVTZ are used for the YAM100 and QM9-ES data sets, respectively.

## 4 Conclusions

In this work, we have integrated cost-effective *ϵV* ^H^ *V* ^P^ images with rVGG models to predict excited-state energies. The use of the SAD initial guess technique and the semiempirical GFN2-xTB method substantially reduces computational costs compared to fully convergent mean-field calculations. Hyperparameter optimization yields optimal rVGG­(SAD) and rVGG­(GFN2-xTB) models with comparable *L*_1_ deviations of 0.088 and 0.093 eV, respectively, for small organic molecules in the QM9-40K data set. Transferability assessments confirm the superior generalization ability of the rVGG­(GFN2-xTB) model. When applied to the practical CSD data set, divergent performances are observed, with *L*_1_ deviations of 0.186 eV for rVGG­(SAD) and 0.130 eV for rVGG­(GFN2-xTB), which can be attributed to the inherent randomness associated with SAD guess orbitals. The optimal rVGG­(GFN2-xTB) model trained on the CSD data set demonstrates promising results when extended to a broader YAM100 data set containing organic photofunctional materials beyond OSCs. Perturbation analysis underscores the significance of frontier orbital energies for the rVGG model, motivating the use of transfer learning by initializing the rVGG­(GFN2-xTB) model with weights from a pretrained rVGG­(PBE0) model. This strategy leads to improved prediction accuracy, consistent with insights from the perturbation analysis. The cross-comparison with semiempirical excited-state methods suggests that the rVGG­(GFN2-xTB) model achieves sufficient accuracy for practical applications, such as estimating the emission energies of boron­(III)-based TADF emitters. The present work highlights the potential of cost-effective *ϵV* ^H^ *V* ^P^ descriptors for efficient and accurate prediction of excited-state energies and provides valuable guidance for further advancement within a cost-effective excited-state ML framework.

## Supplementary Material





## Data Availability

The data and model files are available free of charge at Materials Cloud (10.24435/materialscloud:4v-ce).

## Notes

Performance of rVGG­(SAD) and rVGG­(GFN2-xTB) models; hyperparameter tuning results; perturbation analysis results; comparison with semiempirical excited-state methods (PDF)
Cartesian coordinates of molecules in the YAM100 and QM9-ES data sets (XYZ)
