# Supplementary material for: Cost-Effective Multi-Channel MolOrbImage for Machine-Learned Excited-State Properties of Practical Photofunctional Materials
Source: J Chem Theory Comput. 2026 Jan 26;22(3):1338–49. doi: 10.1021/acs.jctc.5c01721 (PMC12895422; doi:10.1021/acs.jctc.5c01721)
Supplement: Supplementary file 1 [file ct5c01721_si_001.pdf]

Supporting Information

Cost-Effective Multi-Channel MolOrbImage for Machine-Learned Excited-State Properties of Practical Photofunctional Materials

Ziyong Chen,*,† Jonathan Lam,†,‡ and Vivian Wing-Wah Yam*,†,‡

†*Institute of Molecular Functional Materials and Department of Chemistry, The University of Hong Kong, Hong Kong 999077, P. R. China*

‡*Hong Kong Quantum AI Lab Limited, Hong Kong 999077, P. R. China*

E-mail: wwyam@hku.hk; zyjchen@hku.hk

Supplementary tables

Table S1: Structures of 100 organic photofunctional materials in the YAM100 data set.

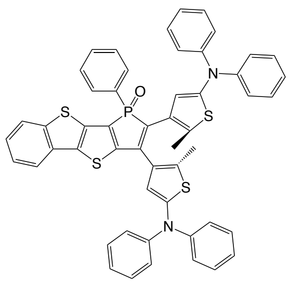

1[1]

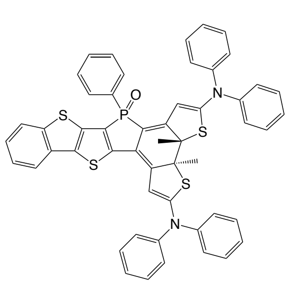

2[1]

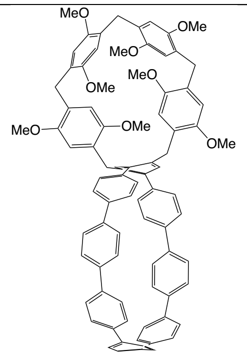

3[2]

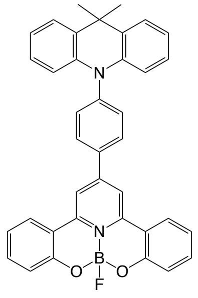

4[3]

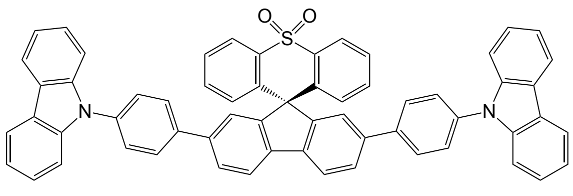

5[4]

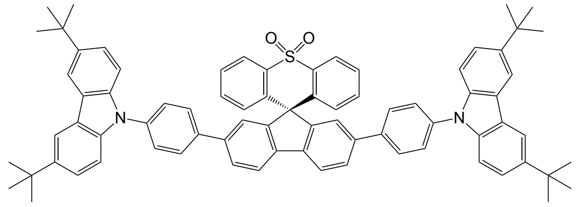

6[4]

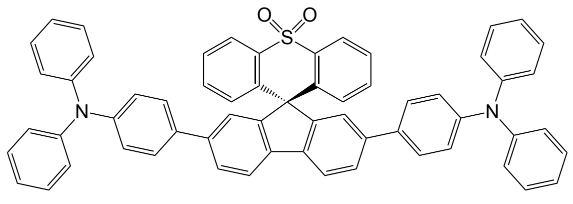

7[4]

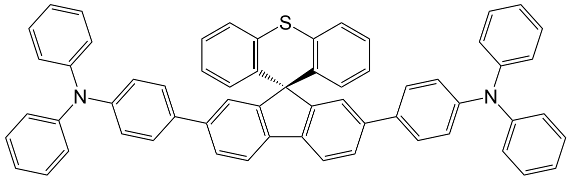

8[4]

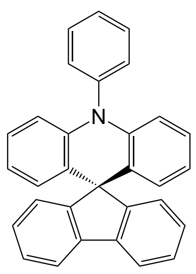

9[5]

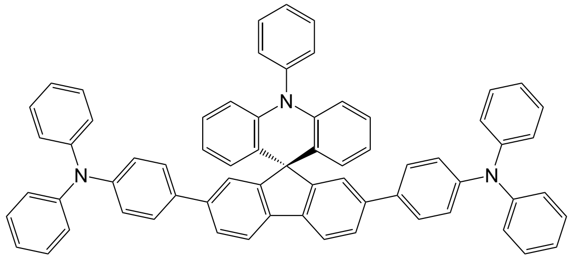

10[5]

Table S1 – continued

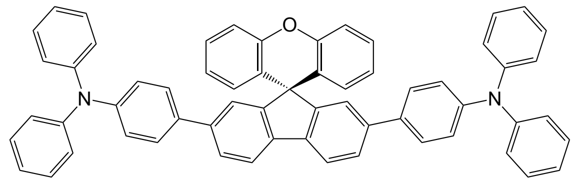

11[5]

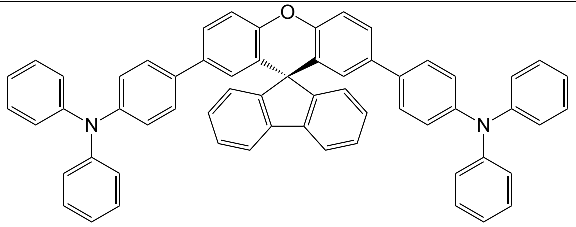

12[5]

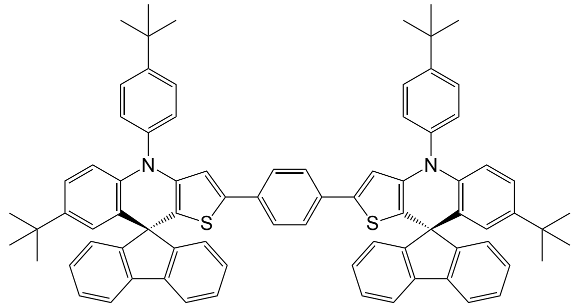

13[6]

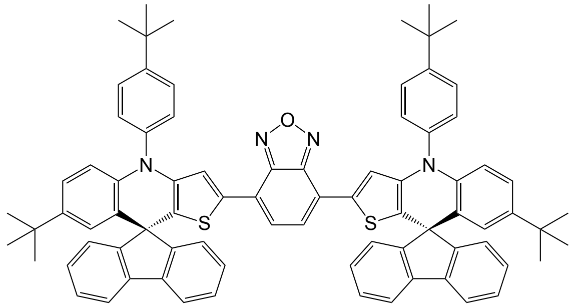

14[6]

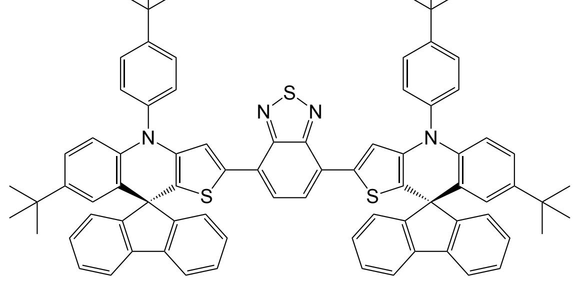

15[6]

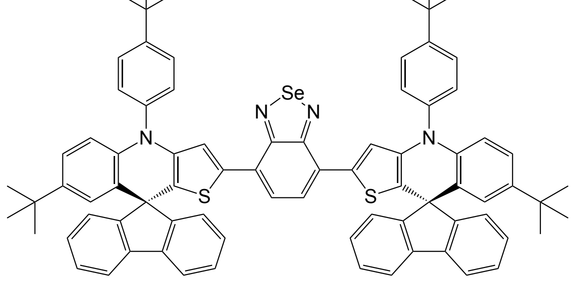

16[6]

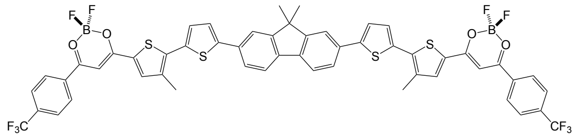

17[7]

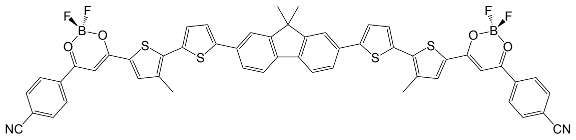

18[7]

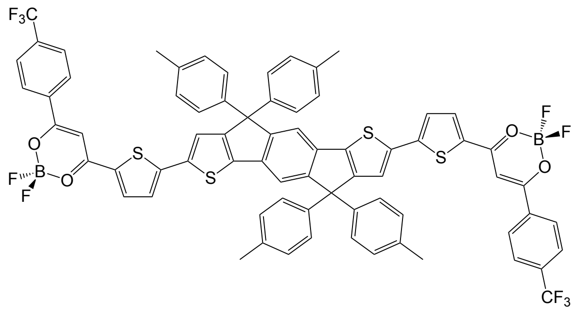

19[7]

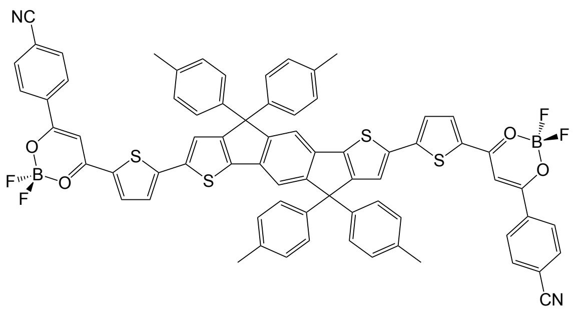

20[7]

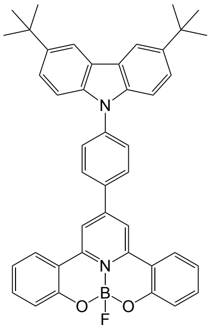

21[3]

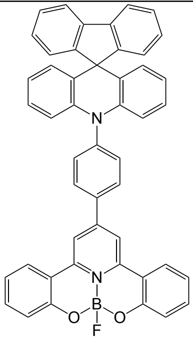

22[3]

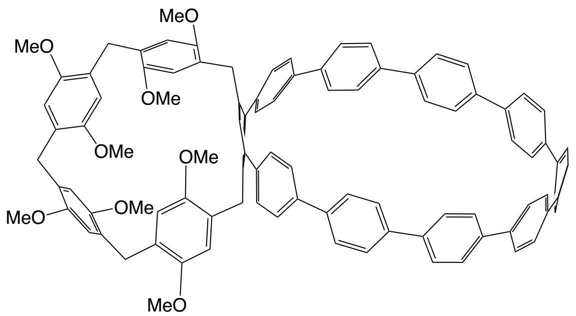

23[2]

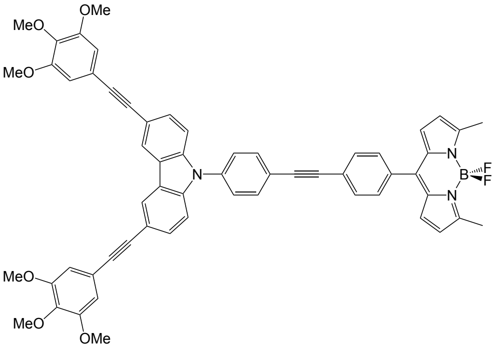

24[8]

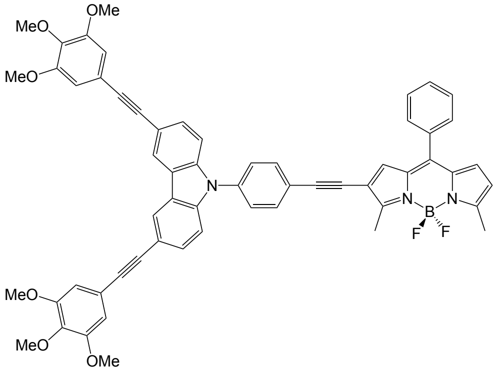

25[8]

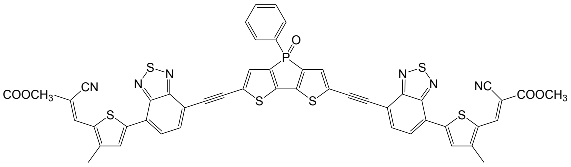

26[9]

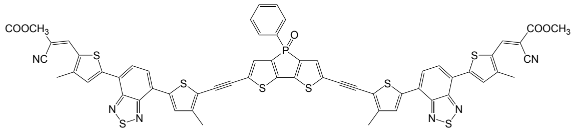

27[9]

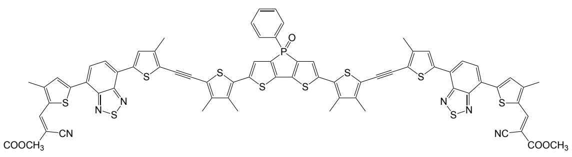

28[9]

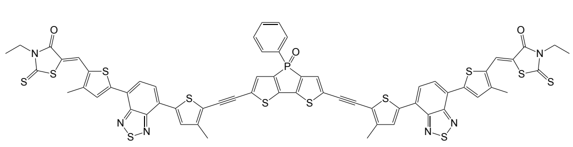

29[9]

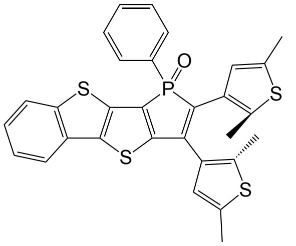

30[1]

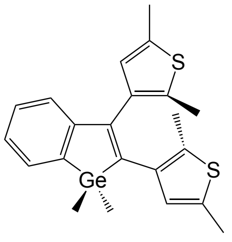

31[10]

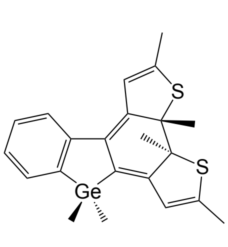

32[10]

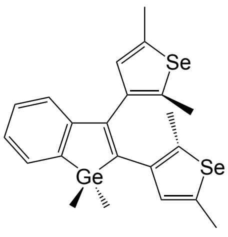

33[10]

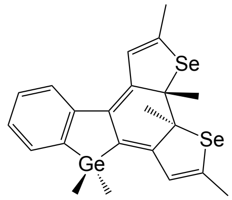

34[10]

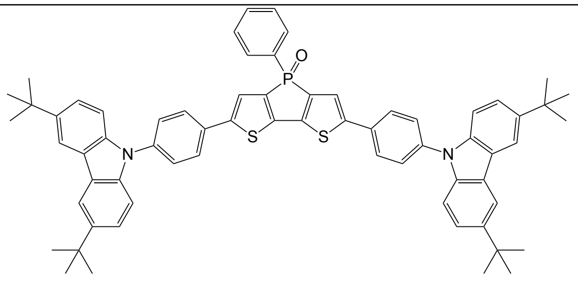

35[11]

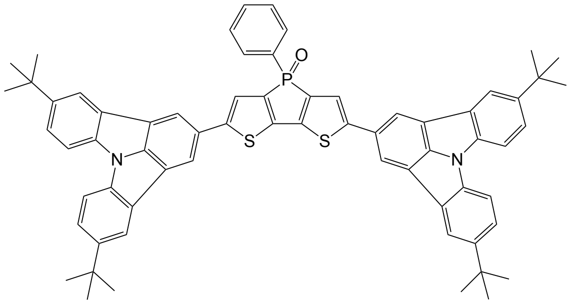

36[11]

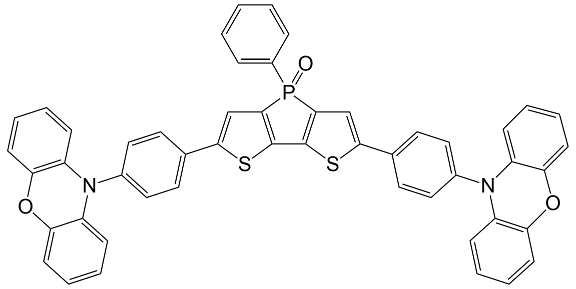

37[11]

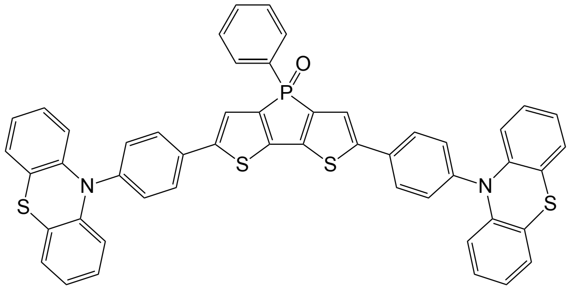

38[11]

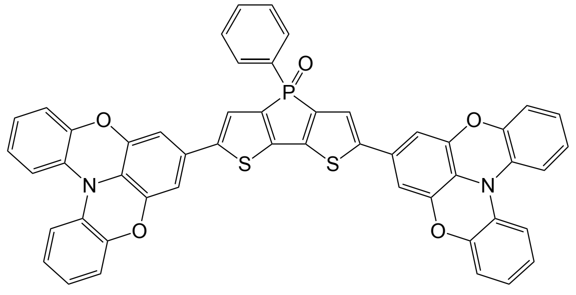

39[11]

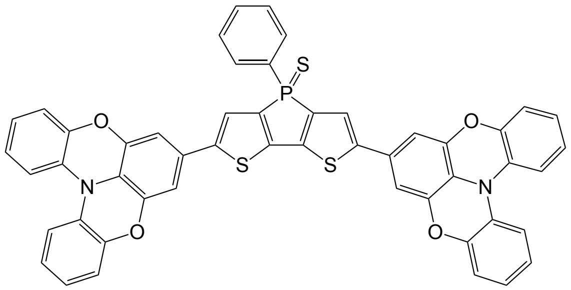

40[11]

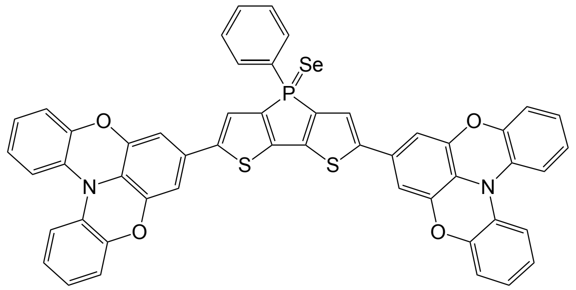

41[11]

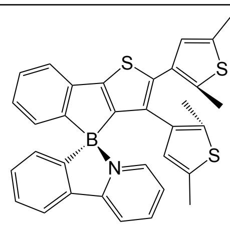	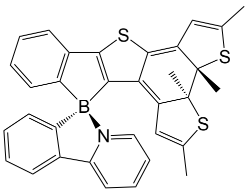	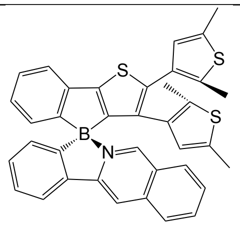	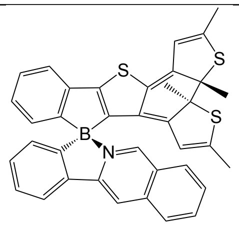
**42**[12]	**43**[12]	**44**[12]	**45**[12]
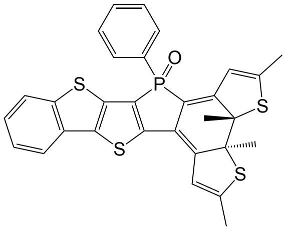	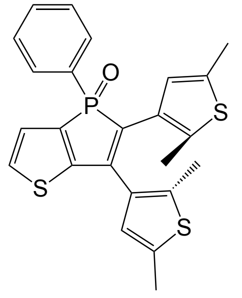	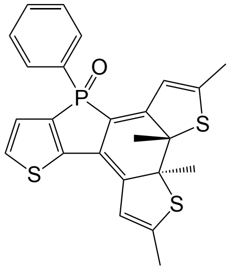	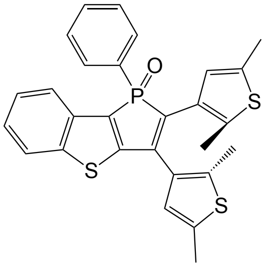
**46**[1]	**47**[1]	**48**[1]	**49**[1]
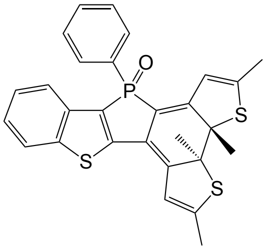	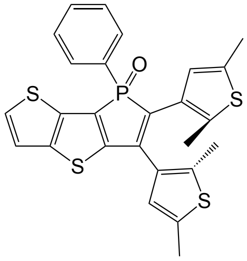	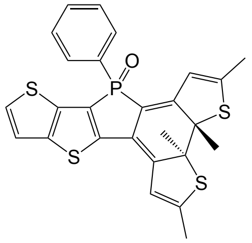	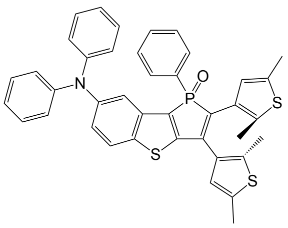
**50**[1]	**51**[1]	**52**[1]	**53**[1]
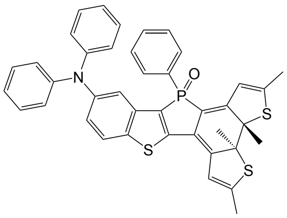	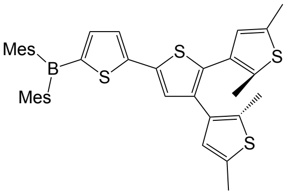	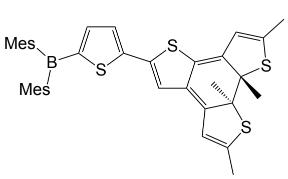	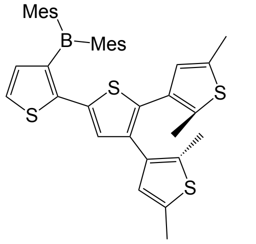
**54**[1]	**55**[13]	**56**[13]	**57**[13]
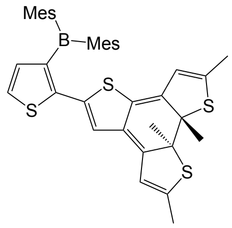		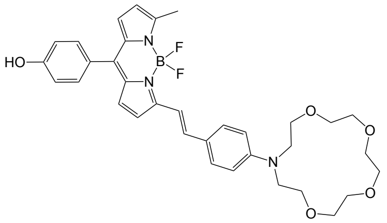	
**58**[13]		**59**[14]	

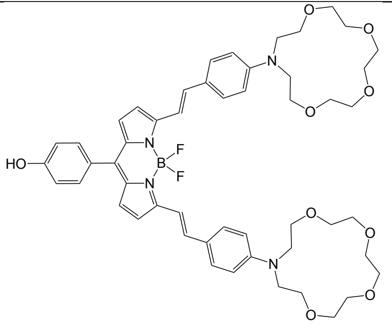

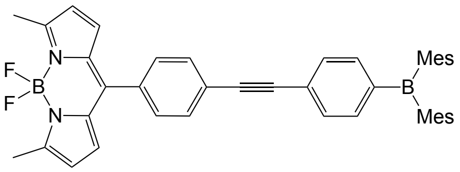

60[14] **61**[14]

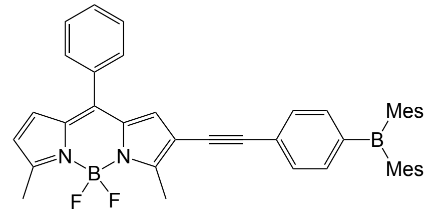

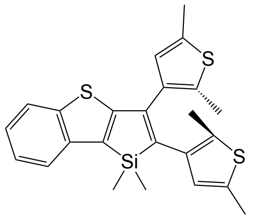

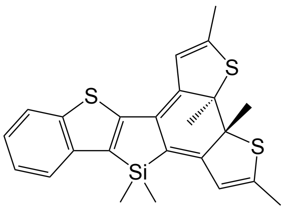

62[14] **63**[15] **64**[15]

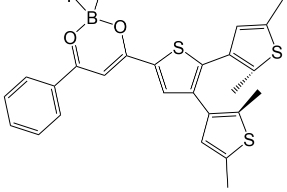

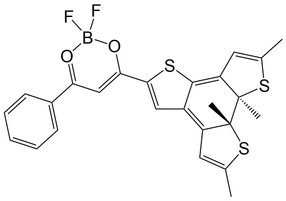

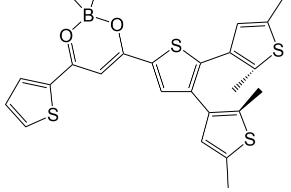

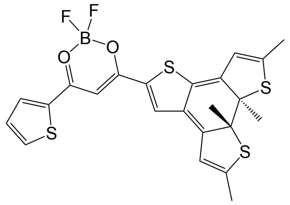

65[16] **66**[16] **67**[16] **68**[16]

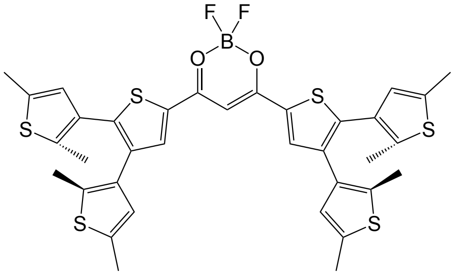

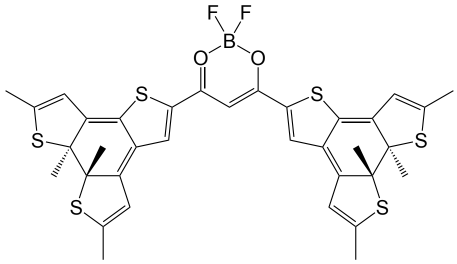

69[16] **70**[16]

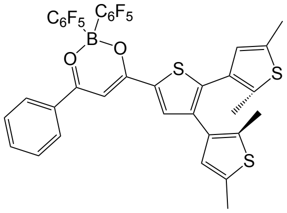

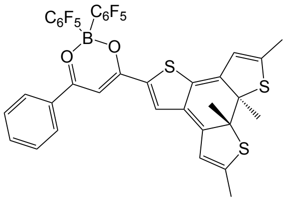

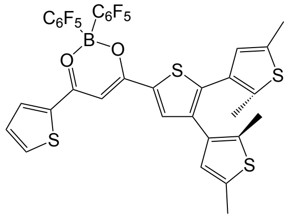

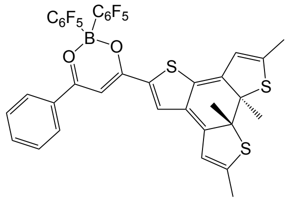

71[16] **72**[16] **73**[16] **74**[16]

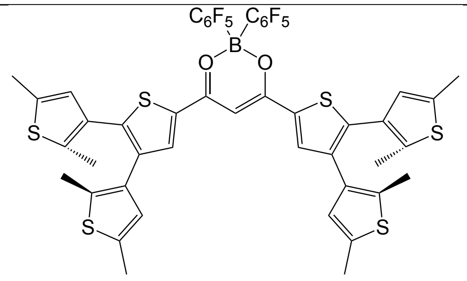

75[16]

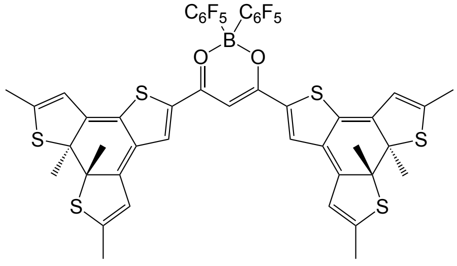

76[16]

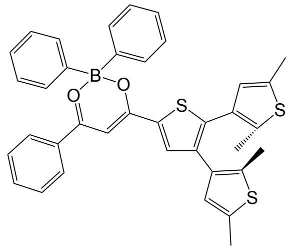

77[16]

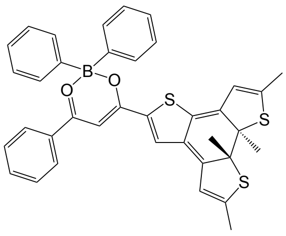

78[16]

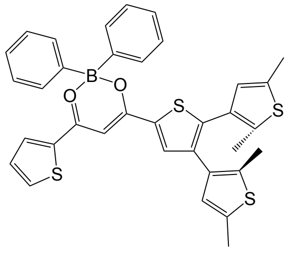

79[16]

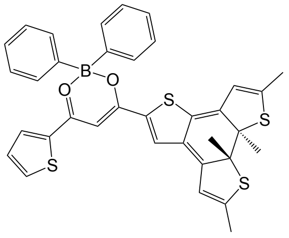

80[16]

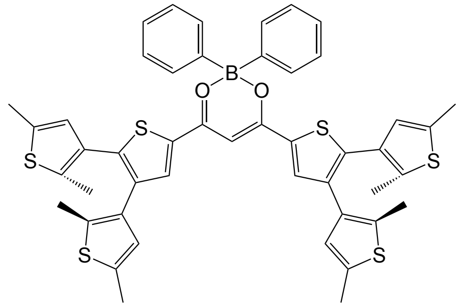

81[16]

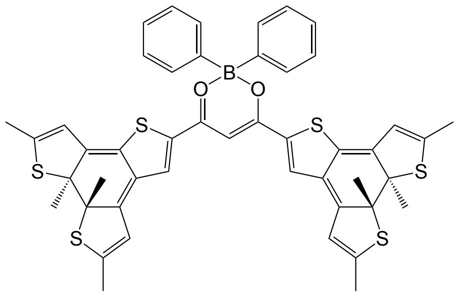

82[16]

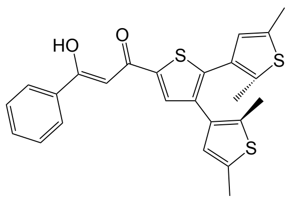

83[16]

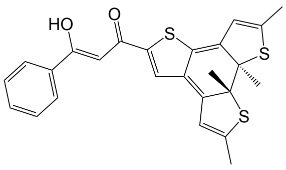

84[16]

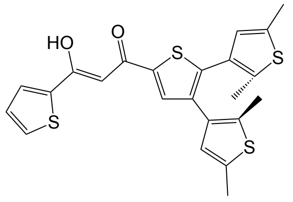

85[16]

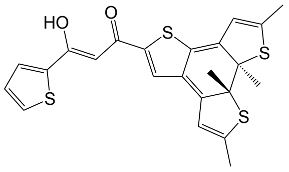

86[16]

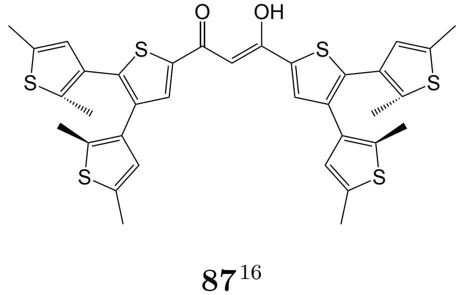

87[16]

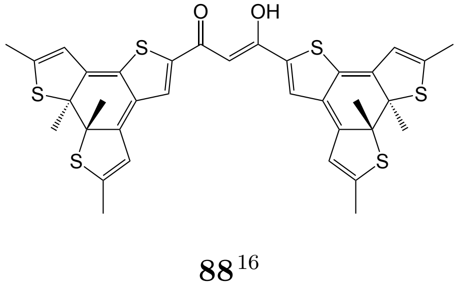

88[16]

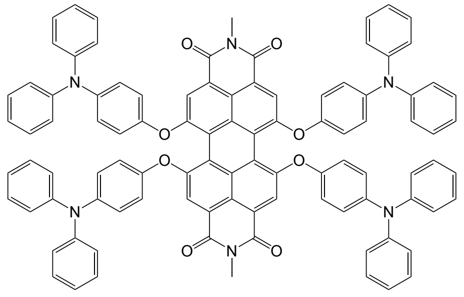

89[17]

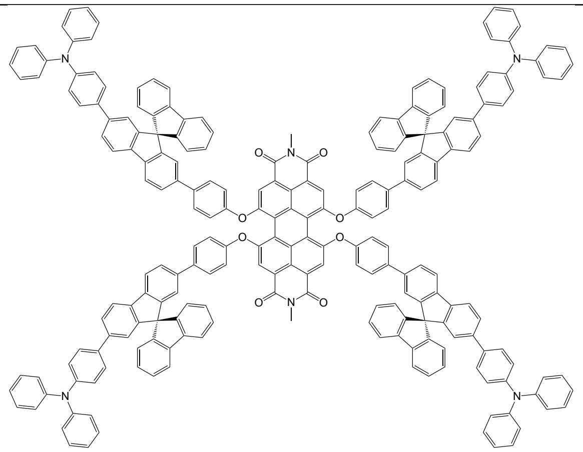

90[17]

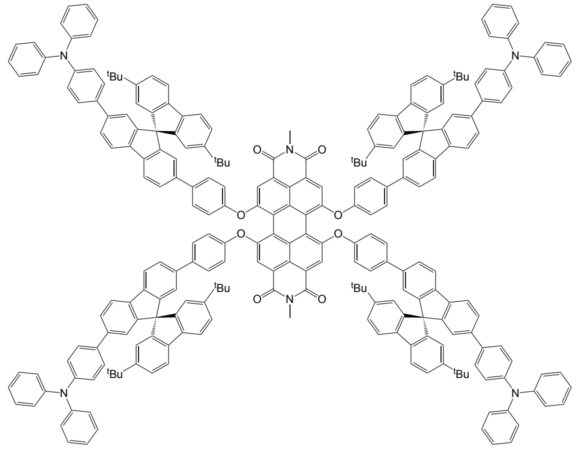

91[17]

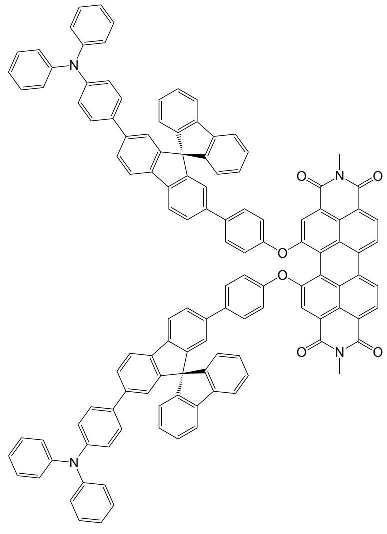

92[17]

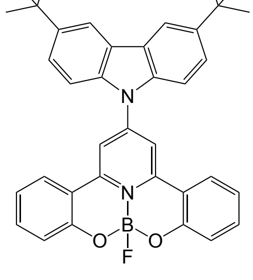

93[18]

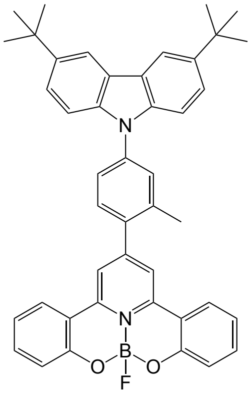

94[18]

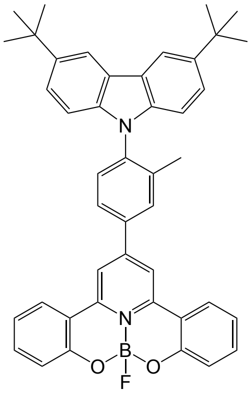

95[18]

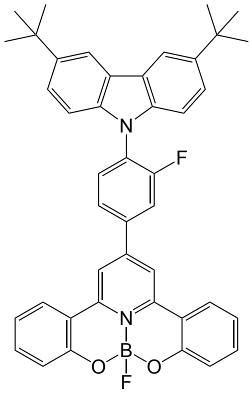

96[18]

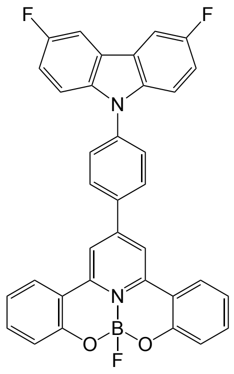	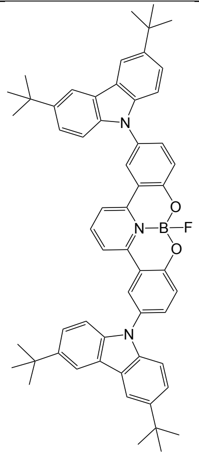	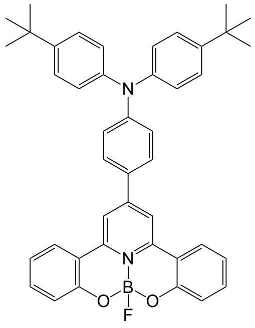	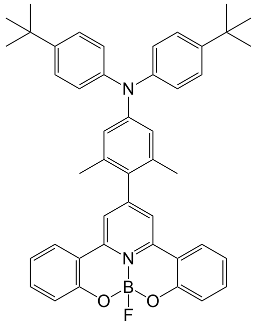
**97**[18]	**98**[18]	**99**[18]	**100**[18]

Table S2: Statistics of number of basis functions (N_{BF}) and electrons (N_{Elec}) for molecules in the QM9-40K and CSD data sets at the SAD/PBE0 and GFN2-xTB levels.

Data set	Method		Max	Min	Median
QM9-40K	SAD/PBE0/cc-pVTZ	N_{BF}	522	120	368
		N_{Elec}	72	22	62
	SAD/PBE0/STO-3G	N_{BF}	63	18	50
		N_{Elec}	72	22	62
	GFN2-xTB	N_{BF}	54	15	42
		N_{Elec}	54	16	46
CSD	SAD/PBE0/def2-SVP	N_{BF}	5,536	78	486
		N_{Elec}	2,088	42	202
	GFN2-xTB	N_{BF}	1,472	26	134
		N_{Elec}	1,480	24	138

Table S3: L_1 deviations (eV) in predicting excited-state properties computed at the ADC(2)/cc-pVTZ level using $\epsilon V^{\mathrm{H}} V^{\mathrm{P}}$+rVGG models ($N_{\mathrm{train}}$ = 32,000) for the QM9-40K data set. In the construction of $\epsilon V^{\mathrm{H}} V^{\mathrm{P}}$ images, SAD/PBE0/cc-pVTZ and GFN2-xTB computed molecular orbitals have been used.

β (Hartree^{-1})	0.1	1	10	100	1000
rVGG(SAD)					
$N_{\mathrm{MO}} = 20$	0.121	0.131	0.140	0.134	0.140
$N_{\mathrm{MO}} = 40$	0.113	0.104	0.103	0.106	0.117
$N_{\mathrm{MO}} = 60$	0.104	0.103	0.096	0.101	0.120
$N_{\mathrm{MO}} = 80$	0.095	0.094	0.088	0.096	0.096
rVGG(GFN2-xTB)					
$N_{\mathrm{MO}} = 20$	0.119	0.127	0.124	0.130	0.138
$N_{\mathrm{MO}} = 40$	0.108	0.107	0.119	0.114	0.111
$N_{\mathrm{MO}} = 60$	0.099	0.103	0.093	0.099	0.098

Table S4: Mean (μ) and standard deviation (σ) values (eV) of errors in predicting excited-state properties computed at the ADC(2)/cc-pVTZ level using the optimal $\epsilon V^{\mathrm{H}} V^{\mathrm{P}}$+rVGG models ($N_{\mathrm{train}}$ = 32,000) for the QM9-40K data set.

	rVGG(SAD)[a]		rVGG(GFN2-xTB)[b]		rVGG(PBE0)[c]	
	μ	σ	μ	σ	μ	σ
E_{S_1}	0.005	0.118	0.015	0.124	0.007	0.067
E_{S_2}	0.000	0.139	0.004	0.140	0.002	0.095
E_{S_3}	0.005	0.144	0.009	0.141	0.003	0.108
E_{T_1}	0.004	0.099	0.012	0.106	0.001	0.059
E_{T_2}	0.007	0.121	0.009	0.125	0.000	0.089
E_{T_3}	0.002	0.131	0.010	0.133	0.006	0.106
$\Delta E_{\mathrm{S}_1-\mathrm{T}_1}$	0.001	0.093	0.003	0.098	0.006	0.077
$\Delta E_{\mathrm{S}_2-\mathrm{S}_1}$	−0.005	0.162	−0.011	0.167	−0.006	0.117
$\Delta E_{\mathrm{T}_2-\mathrm{T}_1}$	0.003	0.142	−0.003	0.148	−0.001	0.108

[a] $\beta = 10$ Hartree^{-1} and $N_{\mathrm{MO}} = 80$.
[b] $\beta = 10$ Hartree^{-1} and $N_{\mathrm{MO}} = 60$.
[c] $\beta = 100$ Hartree^{-1} and $N_{\mathrm{MO}} = 80$, taken from ref 19.

Table S5: Directional exceedance probabilities (P) for errors in predicting excited-state energies computed at the ADC(2)/cc-pVTZ level using the optimal $\epsilon V^{\mathrm{H}} V^{\mathrm{P}}$+rVGG models ($N_{\mathrm{train}} = 32{,}000$) for the QM9-40K data set. Here, P^+ and P^- represent $P(\text{error} > t)$ and $P(\text{error} < -t)$, respectively, and $t = 0.1$ eV.

	rVGG(SAD)		rVGG(GFN2-xTB)		rVGG(PBE0)	
	P^+	P^-	P^+	P^-	P^+	P^-
E_{S_1}	0.135	0.158	0.135	0.195	0.041	0.069
E_{S_2}	0.184	0.198	0.195	0.211	0.104	0.112
E_{S_3}	0.192	0.218	0.192	0.228	0.131	0.151
E_{T_1}	0.096	0.111	0.100	0.144	0.038	0.044
E_{T_2}	0.135	0.168	0.153	0.183	0.099	0.094
E_{T_3}	0.161	0.185	0.153	0.200	0.114	0.144

Table S6: Mean absolute errors (MAEs, eV) and Pearson correlation coefficients (r) in predicting excited-state properties computed at the ADC(2)/cc-pVTZ level using the optimal $\epsilon V^{\mathrm{H}} V^{\mathrm{P}}$+rVGG models ($N_{\mathrm{train}} = 32{,}000$) for the QM9-40K data set.

	rVGG(SAD)		rVGG(GFN2-xTB)		rVGG(PBE0)	
	MAE	r	MAE	r	MAE	r
E_{S_1}	0.082	0.995	0.090	0.995	0.047	0.998
E_{S_2}	0.099	0.991	0.104	0.990	0.068	0.996
E_{S_3}	0.104	0.987	0.107	0.988	0.078	0.993
E_{T_1}	0.066	0.997	0.074	0.997	0.042	0.999
E_{T_2}	0.083	0.994	0.090	0.994	0.063	0.997
E_{T_3}	0.092	0.992	0.095	0.992	0.075	0.995
$\Delta E_{\mathrm{S}_1-\mathrm{T}_1}$	0.062	0.988	0.066	0.987	0.051	0.992
$\Delta E_{\mathrm{S}_2-\mathrm{S}_1}$	0.121	0.965	0.125	0.962	0.086	0.982
$\Delta E_{\mathrm{T}_2-\mathrm{T}_1}$	0.101	0.974	0.107	0.972	0.078	0.985

Table S7: MAE(eV) and r from rVGG(SAD) predictions based on SAD/PBE0 orbitals using STO-3G and cc-pVTZ basis sets for the QM9-40K data set.

		E_{S_1}	E_{S_2}	E_{S_3}	E_{T_1}	E_{T_2}	E_{T_3}	$\Delta E_{S_1-T_1}$	$\Delta E_{S_2-S_1}$	$\Delta E_{T_2-T_1}$
$\beta = 10$ Hartree^{-1} and $N_{MO} = 80$										
STO-3G	MAE	0.088	0.104	0.109	0.070	0.087	0.098	0.065	0.121	0.103
	r	0.994	0.989	0.986	0.997	0.994	0.991	0.987	0.965	0.974
cc-pVTZ	MAE	0.082	0.099	0.104	0.066	0.083	0.092	0.062	0.121	0.101
	r	0.995	0.991	0.987	0.997	0.994	0.992	0.988	0.965	0.974
Absolute	MAE	0.006	0.005	0.005	0.004	0.004	0.005	0.002	0.000	0.002
deviation	r	0.001	0.001	0.002	0.000	0.001	0.001	0.001	0.000	0.000
$\beta = 10$ Hartree^{-1} and $N_{MO} = 60$										
STO-3G	MAE	0.090	0.106	0.112	0.075	0.092	0.099	0.072	0.126	0.111
	r	0.994	0.989	0.985	0.996	0.993	0.991	0.985	0.961	0.970
cc-pVTZ	MAE	0.091	0.109	0.112	0.072	0.091	0.100	0.072	0.130	0.110
	r	0.994	0.989	0.985	0.997	0.993	0.991	0.984	0.960	0.972
Absolute	MAE	0.001	0.003	0.000	0.003	0.001	0.000	0.001	0.004	0.001
deviation	r	0.000	0.001	0.000	0.000	0.000	0.000	0.000	0.001	0.003

Table S8: L_1 deviations (eV) in predicting excited-state properties computed at the ADC(2)/cc-pVTZ level using $\epsilon V^{H} V^{P}$+rVGG models (N_{train} = 32,000) for the QM9-ES data set in ref 20.

N_{MO}	20	40	60	80
rVGG(SAD)^a	0.581	0.618	0.597	0.819
rVGG(GFN2-xTB)^a	0.297	0.319	0.340	
rVGG(PBE0)^b	0.185	0.183	0.260	0.750

$^a\beta = 10$ Hartree^{-1}.
$^b\beta = 100$ Hartree^{-1}.

Table S9: MAEs (eV) and r in predicting $S_0 \rightarrow S_n$ ($n = 1-3$) transition energies computed at the ADC(2)/cc-pVTZ level using $\epsilon V^{H} V^{P}$+rVGG models (N_{train} = 32,000) for the QM9-ES data set.

	rVGG(SAD)^a		rVGG(GFN2-xTB)^a		rVGG(PBE0)^b	
	MAE	r	MAE	r	MAE	r
E_{S_1}	0.490	0.911	0.321	0.961	0.143	0.993
E_{S_2}	0.665	0.861	0.289	0.942	0.160	0.978
E_{S_3}	0.678	0.789	0.280	0.931	0.187	0.972
E_{T_1}	0.526	0.901	0.255	0.965	0.172	0.984
E_{T_2}	0.513	0.900	0.337	0.945	0.205	0.981
E_{T_3}	0.611	0.843	0.300	0.929	0.230	0.956

$^a\beta = 10$ Hartree^{-1} and $N_{\text{MO}} = 20$.
$^b\beta = 100$ Hartree^{-1} and $N_{\text{MO}} = 40$.

Table S10: L_1 deviations (eV) in predicting excited-state properties computed at the TDA/PBE0/def2-SVP level using $\epsilon V^{H} V^{P}$+rVGG models (N_{train} = 40,000) for the CSD data set. In the construction of $\epsilon V^{H} V^{P}$ images, SAD/PBE0/def2-SVP and GFN2-xTB computed molecular orbitals have been used.

β (Hartree^{-1})	0.1	1	10	100	1000
rVGG(SAD)					
$N_{\text{MO}} = 20$	0.232	0.231	0.220	0.227	0.231
$N_{\text{MO}} = 40$	0.218	0.225	0.195	0.224	0.208
$N_{\text{MO}} = 60$	0.200	0.198	0.192	0.201	0.187
$N_{\text{MO}} = 80$	0.208	0.200	0.188	0.191	0.197
$N_{\text{MO}} = 100$	0.208	0.202	0.194	0.186	0.195
rVGG(GFN2-xTB)					
$N_{\text{MO}} = 20$	0.176	0.170	0.172	0.159	0.166
$N_{\text{MO}} = 40$	0.159	0.164	0.170	0.138	0.147
$N_{\text{MO}} = 60$	0.148	0.147	0.141	0.134	0.147
$N_{\text{MO}} = 80$	0.146	0.145	0.145	0.133	0.131
$N_{\text{MO}} = 100$	0.147	0.150	0.145	0.130	0.133

Table S11: MAEs (eV) and r in predicting excited-state properties computed at the PBE0/def2-SVP level using the optimal $\epsilon V^{\mathrm{H}} V^{\mathrm{P}}$+rVGG models ($N_{\mathrm{train}}$ = 40,000) for the CSD data set.

	rVGG(SAD)[a]		rVGG(GFN2-xTB)[a]		rVGG(PBE0)[b]	
	MAE	r	MAE	r	MAE	r
E_{S_1}	0.202	0.796	0.121	0.941	0.057	0.985
E_{S_2}	0.195	0.808	0.133	0.929	0.070	0.979
E_{S_3}	0.193	0.825	0.137	0.926	0.080	0.976
E_{T_1}	0.172	0.861	0.122	0.940	0.072	0.978
E_{T_2}	0.183	0.829	0.133	0.922	0.088	0.963
E_{T_3}	0.171	0.821	0.134	0.906	0.093	0.954
$\Delta E_{\mathrm{S}_1-\mathrm{T}_1}$	0.168	0.834	0.126	0.909	0.085	0.958
$\Delta E_{\mathrm{S}_2-\mathrm{S}_1}$	0.182	0.650	0.142	0.817	0.089	0.920
$\Delta E_{\mathrm{T}_2-\mathrm{T}_1}$	0.180	0.774	0.154	0.849	0.106	0.931

[a] β = 100 Hartree^{-1} and N_{MO} = 100.
[b] β = 100 Hartree^{-1} and N_{MO} = 80, taken from ref 19.

Table S12: Application of the optimal rVGG models to 100 organic photofunctional materials in the YAM100 data set. Results (eV) are for singlet excited states.

Compound	rVGG(GFN2-XTB)			rVGG(PBE0)			TDA/PBE0		
	E_{S_1}	E_{S_2}	E_{S_3}	E_{S_1}	E_{S_2}	E_{S_3}	E_{S_1}	E_{S_2}	E_{S_3}
**1**	2.755	3.046	3.428	2.624	2.914	3.215	2.586	2.791	3.196
**2**	2.521	3.051	3.257	2.483	2.861	3.239	2.394	2.877	3.249
**3**	3.061	3.494	3.548	2.910	3.170	3.314	2.843	3.271	3.511
**4**	2.505	3.180	3.618	2.558	3.065	3.375	2.581	3.158	3.271
**5**	3.249	3.492	3.859	3.371	3.623	3.745	3.334	3.545	3.817
**6**	3.181	3.329	3.777	3.190	3.387	3.646	3.200	3.393	3.671
**7**	3.292	3.534	3.833	3.098	3.304	3.530	3.121	3.422	3.472
**8**	3.423	3.582	3.827	3.347	3.522	3.801	3.237	3.498	3.779
**9**	3.242	3.695	3.791	3.674	3.750	3.936	3.759	3.875	3.995
**10**	3.449	3.667	3.727	3.350	3.515	3.599	3.298	3.374	3.563
**11**	3.495	3.617	3.857	3.321	3.496	3.850	3.264	3.519	3.906
**12**	3.547	3.580	3.751	3.563	3.631	3.778	3.617	3.703	3.801
**13**	3.194	3.260	3.488	3.104	3.328	3.573	3.168	3.290	3.472
**14**	2.054	2.418	2.901	2.073	2.298	2.901	2.105	2.251	2.831
**15**	2.087	2.435	2.848	2.039	2.172	2.784	2.034	2.207	2.729
**16**	1.930	2.243	2.823	1.888	2.089	2.599	1.898	2.088	2.560
**17**	2.595	2.742	3.191	2.480	2.647	3.048	2.434	2.616	3.035
**18**	2.343	2.596	3.004	2.381	2.590	2.959	2.366	2.530	2.960
**19**	2.341	2.643	2.797	2.180	2.547	2.965	2.187	2.486	3.036
**20**	2.273	2.498	2.751	2.149	2.531	2.884	2.132	2.409	2.974
**21**	2.987	3.310	3.632	3.063	3.246	3.493	3.051	3.181	3.585
**22**	2.532	3.141	3.488	2.646	3.240	3.328	2.690	3.154	3.384
**23**	2.980	3.312	3.359	2.782	2.996	3.235	3.154	3.208	3.401
**24**	2.447	2.665	3.055	2.434	2.571	2.686	2.456	2.893	3.180
**25**	2.519	2.717	3.088	2.258	2.445	2.663	2.237	2.706	2.776
**26**	1.975	2.221	2.626	2.126	2.337	2.584	1.979	2.288	2.596
**27**	1.940	2.220	2.467	1.852	2.225	2.409	1.829	2.038	2.368
**28**	1.840	2.112	2.376	1.811	2.050	2.150	1.826	1.936	2.165
**29**	2.124	2.311	2.373	1.850	2.034	2.195	1.802	2.019	2.249
**30**	3.192	3.541	3.774	3.101	3.498	3.628	3.085	3.544	3.592
**31**	3.715	4.142	4.263	3.779	4.231	4.379	3.738	4.203	4.447
**32**	2.932	3.519	3.809	2.881	3.529	3.709	2.741	3.479	3.858
**33**	3.547	4.063	4.220	3.682	4.167	4.248	3.624	4.125	4.312
**34**	2.779	3.457	3.729	2.689	3.365	3.479	2.606	3.343	3.388
**35**	2.826	3.151	3.584	2.815	2.916	3.425	2.774	3.021	3.420
**36**	3.110	3.319	3.671	3.063	3.352	3.469	3.014	3.362	3.374
**37**	2.523	2.629	3.321	2.257	2.301	3.128	2.274	2.288	3.155
**38**	2.508	2.707	3.198	2.522	2.586	3.140	2.544	2.544	3.143
**39**	2.466	2.744	3.201	2.554	2.698	3.485	2.525	2.747	3.449
**40**	2.314	2.558	2.802	2.542	2.694	3.250	2.523	2.736	3.177
**41**	2.416	2.566	2.878	2.518	2.673	3.002	2.504	2.726	2.936
**42**	2.788	3.223	3.404	2.540	3.069	3.164	2.491	3.044	3.127
**43**	1.887	2.334	2.735	1.396	1.850	2.186	1.468	2.020	2.345
**44**	2.343	2.960	3.277	2.037	2.632	3.093	2.059	2.697	3.152
**45**	1.533	2.346	2.844	0.823	2.074	2.370	1.027	2.138	2.327
**46**	2.713	3.077	3.458	2.785	3.148	3.428	2.639	3.135	3.475
**47**	3.341	3.695	3.909	3.160	3.675	3.845	3.255	3.819	3.897
**48**	2.674	3.228	3.701	2.761	3.386	3.659	2.706	3.295	3.709

Table S12 – continued

**49**	3.111	3.617	3.828	3.094	3.676	3.726	3.110	3.691	3.717
**50**	2.710	3.087	3.560	2.717	3.220	3.530	2.684	3.206	3.634
**51**	3.104	3.636	3.801	3.137	3.667	3.764	3.116	3.685	3.753
**52**	2.766	3.253	3.609	2.774	3.291	3.553	2.680	3.173	3.600
**53**	2.915	3.464	3.749	2.760	3.139	3.670	2.781	3.112	3.692
**54**	2.924	3.048	3.373	2.548	2.846	3.268	2.655	2.778	3.189
**55**	3.155	3.522	3.658	3.174	3.436	3.599	3.168	3.513	3.598
**56**	2.296	2.738	3.152	1.795	2.684	3.169	1.987	2.687	3.073
**57**	3.367	3.613	3.780	3.260	3.549	3.818	3.221	3.662	3.739
**58**	2.126	2.612	3.136	1.765	2.569	3.133	2.030	2.513	3.081
**59**	2.429	3.191	3.507	2.445	3.153	3.511	2.474	3.208	3.456
**60**	2.211	2.600	3.078	2.194	2.595	3.203	2.149	2.587	3.192
**61**	2.879	3.093	3.256	2.896	3.111	3.337	3.049	3.176	3.289
**62**	2.770	3.122	3.252	2.646	3.058	3.220	2.637	3.154	3.294
**63**	3.309	3.750	3.893	3.416	3.906	4.039	3.387	3.901	3.994
**64**	2.828	3.305	3.609	2.790	3.355	3.585	2.709	3.280	3.728
**65**	2.904	3.178	3.510	2.938	3.252	3.623	2.953	3.314	3.618
**66**	1.708	2.467	3.012	1.412	2.609	2.924	1.659	2.616	2.971
**67**	2.917	3.179	3.415	2.862	3.325	3.540	2.908	3.274	3.563
**68**	1.808	2.580	2.888	1.479	2.537	2.940	1.642	2.547	2.952
**69**	2.751	2.899	3.278	2.763	2.977	3.263	2.841	3.018	3.273
**70**	1.639	2.112	2.657	1.208	1.530	2.066	1.492	1.547	2.198
**71**	2.711	2.996	3.235	2.741	3.016	3.150	2.778	3.129	3.152
**72**	1.589	2.437	3.033	1.342	2.586	2.712	1.533	2.563	2.868
**73**	2.669	2.892	3.191	2.702	2.957	3.161	2.749	3.099	3.125
**74**	1.435	2.414	2.860	1.412	2.531	2.684	1.510	2.506	2.837
**75**	2.484	2.938	3.180	2.651	2.798	3.057	2.676	2.835	3.091
**76**	1.378	1.661	2.276	1.157	1.478	2.063	1.375	1.413	2.153
**77**	2.388	2.978	3.073	2.588	2.904	2.940	2.808	2.954	3.022
**78**	1.907	2.512	2.934	1.539	2.304	2.446	1.678	2.580	2.625
**79**	2.479	3.012	3.097	2.612	2.871	3.045	2.828	2.912	2.969
**80**	1.817	2.320	2.658	1.544	2.263	2.368	1.650	2.557	2.566
**81**	2.217	2.816	3.095	2.637	2.808	2.945	2.772	2.839	2.986
**82**	1.476	1.871	2.108	1.410	1.637	1.995	1.506	1.558	2.213
**83**	3.103	3.410	3.690	3.290	3.474	4.022	3.308	3.694	3.922
**84**	1.949	2.731	3.196	1.777	2.749	3.175	1.932	2.701	3.174
**85**	3.161	3.481	3.612	3.196	3.514	3.899	3.241	3.624	3.866
**86**	1.833	2.492	3.004	1.781	2.719	3.112	1.901	2.619	3.146
**87**	3.016	3.377	3.478	3.100	3.293	3.551	3.153	3.360	3.615
**88**	1.808	2.109	2.596	1.494	1.839	2.199	1.713	1.800	2.273
**89**	1.812	1.896	2.129	1.751	1.772	2.079	1.816	1.860	1.898
**90**	1.490	1.596	1.605	1.779	1.729	2.075	1.876	1.886	1.924
**91**	1.488	1.615	1.658	1.763	1.710	2.046	1.850	1.870	1.935
**92**	1.619	1.649	1.877	1.545	1.556	1.993	1.718	1.838	2.174
**93**	3.231	3.294	3.452	3.286	3.384	3.573	3.361	3.434	3.631
**94**	3.079	3.410	3.684	3.107	3.306	3.484	3.104	3.251	3.580
**95**	3.030	3.376	3.696	3.002	3.266	3.431	3.023	3.181	3.541
**96**	3.016	3.289	3.640	2.958	3.196	3.412	2.969	3.128	3.434
**97**	3.102	3.403	3.604	3.126	3.197	3.512	3.139	3.225	3.579
**98**	2.773	2.799	3.130	2.594	2.672	3.032	2.658	2.722	2.956
**99**	3.037	3.365	3.815	3.075	3.279	3.499	3.077	3.242	3.499
**100**	2.932	3.380	3.652	2.986	3.221	3.373	2.907	3.204	3.374

Table S13: Application of the optimal rVGG models to 100 organic photofunctional materials in the YAM100 data set. Results (eV) are for triplet excited states.

Compound	rVGG(GFN2-XTB)			rVGG(PBE0)			TDA/PBE0		
	E_{T_1}	E_{T_2}	E_{T_3}	E_{T_1}	E_{T_2}	E_{T_3}	E_{T_1}	E_{T_2}	E_{T_3}
**1**	2.205	2.650	2.885	2.161	2.676	2.805	1.928	2.545	2.640
**2**	1.503	2.483	2.808	1.458	2.411	2.807	1.451	2.179	2.810
**3**	2.448	2.780	3.043	2.345	2.646	2.765	2.359	2.652	2.677
**4**	2.268	2.816	3.064	2.446	2.739	2.984	2.574	2.678	2.859
**5**	2.629	3.120	3.314	2.765	3.150	3.290	2.755	3.183	3.429
**6**	2.710	3.059	3.248	2.719	2.990	3.149	2.721	3.082	3.287
**7**	2.501	3.140	3.256	2.487	2.925	3.080	2.587	2.895	3.314
**8**	2.594	3.051	3.255	2.665	3.163	3.299	2.659	2.949	3.327
**9**	2.963	3.302	3.478	3.283	3.397	3.551	3.314	3.428	3.670
**10**	2.776	3.180	3.280	2.685	2.996	3.080	2.693	2.981	3.335
**11**	2.550	3.052	3.404	2.755	3.050	3.282	2.678	2.959	3.332
**12**	2.879	3.057	3.301	2.942	3.131	3.308	2.980	3.027	3.331
**13**	2.618	2.812	3.047	2.593	2.886	3.132	2.381	2.706	3.144
**14**	1.420	2.128	2.565	1.552	1.936	2.417	1.422	1.956	2.235
**15**	1.435	2.060	2.402	1.551	1.975	2.382	1.437	1.968	2.244
**16**	1.391	1.922	2.285	1.459	1.773	2.238	1.309	1.876	2.126
**17**	1.765	1.928	2.444	1.852	1.950	2.479	1.919	1.975	2.497
**18**	1.579	1.759	2.240	1.836	1.927	2.413	1.882	1.934	2.470
**19**	1.655	1.896	2.152	1.605	1.871	2.390	1.611	1.832	2.428
**20**	1.713	1.915	2.318	1.575	1.824	2.370	1.584	1.795	2.388
**21**	2.417	2.939	3.165	2.740	3.014	3.160	2.701	2.799	2.876
**22**	2.465	2.858	3.039	2.481	2.815	3.009	2.675	2.682	2.857
**23**	2.572	2.818	3.023	2.412	2.538	2.748	2.565	2.781	2.825
**24**	1.754	2.280	2.475	1.653	2.100	2.391	1.609	2.369	2.716
**25**	1.893	2.303	2.566	1.602	2.087	2.395	1.563	2.202	2.544
**26**	1.430	1.534	2.064	1.448	1.659	2.188	1.342	1.478	1.878
**27**	1.361	1.442	1.942	1.343	1.510	2.060	1.307	1.367	1.697
**28**	1.303	1.430	1.810	1.326	1.398	1.846	1.288	1.295	1.764
**29**	1.544	1.666	2.001	1.339	1.470	1.966	1.274	1.317	1.643
**30**	2.156	3.023	3.153	1.962	2.966	3.070	1.996	2.951	3.116
**31**	2.538	3.418	3.642	2.711	3.505	3.733	2.674	3.421	3.456
**32**	1.480	2.622	3.111	1.514	2.735	3.250	1.567	2.638	3.348
**33**	2.462	3.205	3.510	2.547	3.432	3.739	2.569	3.262	3.327
**34**	1.497	2.665	3.022	1.462	2.623	2.960	1.554	2.607	3.127
**35**	2.026	2.587	2.997	2.175	2.713	2.905	1.984	2.763	2.981
**36**	2.072	2.784	3.078	2.155	2.741	2.833	2.054	2.916	2.921
**37**	1.887	2.297	2.716	1.996	2.203	2.533	2.022	2.261	2.293
**38**	2.049	2.277	2.686	2.215	2.473	2.709	2.044	2.539	2.539
**39**	2.016	2.355	2.780	1.924	2.436	2.718	1.887	2.393	2.697
**40**	1.604	2.160	2.495	1.907	2.415	2.739	1.901	2.389	2.676
**41**	1.674	2.131	2.442	1.901	2.414	2.628	1.890	2.380	2.659
**42**	2.626	2.819	2.978	2.474	2.782	3.005	2.450	2.614	3.034
**43**	1.458	2.009	2.177	1.265	1.682	2.059	1.058	1.456	2.015
**44**	2.266	2.645	2.881	1.973	2.448	2.691	2.039	2.561	2.659
**45**	1.498	1.937	2.271	0.885	1.706	2.081	1.015	1.042	2.135
**46**	1.464	2.400	2.757	1.510	2.398	2.802	1.527	2.346	2.961
**47**	2.129	3.207	3.494	2.271	3.109	3.350	2.167	3.208	3.278
**48**	1.494	2.517	2.948	1.475	2.518	3.018	1.569	2.453	3.182

Table S13 – continued

**49**	2.101	3.038	3.286	2.032	2.976	3.253	2.059	3.045	3.230
**50**	1.616	2.507	2.863	1.538	2.459	2.914	1.566	2.402	3.057
**51**	2.021	3.023	3.184	2.054	2.922	3.205	2.022	3.041	3.248
**52**	1.576	2.498	2.922	1.514	2.458	3.024	1.550	2.371	3.066
**53**	2.270	2.976	3.178	2.204	2.785	3.063	2.061	2.528	3.048
**54**	1.716	2.677	2.770	1.828	2.581	2.806	1.560	2.396	2.557
**55**	2.182	2.715	3.099	2.258	2.972	3.177	2.228	3.089	3.234
**56**	1.018	2.130	2.428	1.163	2.084	2.467	0.968	1.966	2.521
**57**	2.589	3.035	3.277	2.937	3.183	3.294	2.907	3.084	3.170
**58**	0.922	2.079	2.436	1.201	1.907	2.372	0.993	1.945	2.489
**59**	1.366	2.348	2.921	1.306	2.344	2.818	1.318	2.285	2.840
**60**	1.137	1.976	2.403	1.129	1.901	2.499	1.140	1.923	2.510
**61**	1.932	2.332	2.649	1.672	2.316	2.631	1.612	2.506	2.956
**62**	1.917	2.465	2.767	1.702	2.255	2.755	1.561	2.371	2.771
**63**	2.157	3.146	3.305	2.196	3.254	3.492	2.209	3.160	3.344
**64**	1.490	2.479	2.963	1.524	2.540	3.060	1.539	2.450	3.141
**65**	2.322	2.806	2.915	2.133	2.691	3.011	2.295	2.836	3.061
**66**	1.001	2.120	2.394	0.821	1.873	2.499	0.737	1.802	2.483
**67**	2.248	2.813	3.006	2.085	2.701	2.984	2.267	2.750	3.008
**68**	0.881	2.038	2.291	0.861	1.774	2.444	0.741	1.764	2.458
**69**	1.919	2.519	2.601	2.091	2.595	2.836	2.182	2.514	2.887
**70**	0.828	1.496	2.166	0.778	0.991	1.599	0.687	0.792	1.594
**71**	1.925	2.596	2.801	2.026	2.619	2.965	2.181	2.701	2.919
**72**	0.897	1.817	2.318	0.894	2.013	2.298	0.654	1.708	2.396
**73**	1.873	2.257	2.578	1.984	2.620	2.911	2.140	2.594	2.877
**74**	1.011	1.837	2.330	0.892	2.012	2.295	0.654	1.683	2.354
**75**	1.784	2.138	2.427	1.926	2.525	2.751	2.064	2.393	2.736
**76**	0.492	1.022	1.742	0.716	0.942	1.599	0.592	0.718	1.495
**77**	2.007	2.334	2.558	1.967	2.461	2.688	2.265	2.669	2.823
**78**	0.947	1.865	2.288	0.960	1.874	2.191	0.758	1.812	2.403
**79**	2.011	2.450	2.688	1.977	2.506	2.714	2.231	2.648	2.840
**80**	0.847	1.811	2.148	1.008	1.835	2.121	0.753	1.770	2.371
**81**	1.831	2.260	2.453	1.994	2.469	2.606	2.144	2.515	2.718
**82**	0.539	1.278	1.569	0.814	1.143	1.605	0.698	0.808	1.606
**83**	2.575	2.958	3.110	2.529	2.972	3.070	2.570	2.828	3.309
**84**	1.030	2.417	2.571	1.028	2.061	2.609	0.889	2.022	2.663
**85**	2.482	2.839	3.132	2.464	2.976	3.108	2.494	2.734	3.272
**86**	0.937	2.183	2.378	1.079	1.930	2.506	0.886	1.971	2.559
**87**	2.183	2.776	2.878	2.340	2.796	3.066	2.345	2.684	3.042
**88**	0.858	1.358	1.983	1.032	1.135	1.700	0.843	0.901	1.755
**89**	1.046	1.549	1.860	1.233	1.706	1.691	1.316	1.755	1.814
**90**	1.105	1.279	1.436	1.255	1.318	1.649	1.461	1.865	1.882
**91**	1.115	1.286	1.458	1.201	1.358	1.683	1.384	1.850	1.868
**92**	1.290	1.558	1.685	1.319	1.464	1.716	1.397	1.724	1.834
**93**	2.529	2.878	3.072	2.806	2.940	3.127	2.855	2.874	3.056
**94**	2.618	3.051	3.216	2.800	3.084	3.211	2.745	2.873	2.910
**95**	2.617	3.033	3.232	2.775	3.010	3.193	2.698	2.865	2.908
**96**	2.398	2.869	3.156	2.655	2.946	3.100	2.658	2.746	2.858
**97**	2.432	3.051	3.115	2.729	2.855	3.008	2.669	2.854	2.950
**98**	2.594	2.659	2.964	2.340	2.491	2.704	2.431	2.478	2.804
**99**	2.218	2.915	3.247	2.735	2.976	3.182	2.589	2.765	2.903
**100**	2.520	2.922	3.275	2.631	2.968	3.180	2.769	2.824	2.890

Table S14: Outliers for the optimal rVGG(GFN2-xTB) model in predicting E_{S_1}.Predictions from rVGG(GFN2-xTB) and rVGG(PBE0) models are compared with the TDA/PBE0 computed values. Number of occupied orbitals (N_{OCC}) is provided for each outlier.

	N_{OCC}	E_{S_1}			
		rVGG(GFN2-xTB)[a]	rVGG(GFN2-xTB)[b]	rVGG(PBE0)[c]	TDA/PBE0
**9**	75	3.242	3.626	3.674	3.759
**29**	200	2.124	2.113	1.850	1.802
**43**	92	1.887	1.293	1.396	1.468
**45**	101	1.533	0.999	0.823	1.027
**56**	109	2.296	1.969	1.795	1.987
**77**	106	2.388	2.508	2.588	2.808
**79**	104	2.479	2.344	2.612	2.828
**81**	140	2.217	2.278	2.637	2.772
**90**	552	1.490	1.457	1.779	1.876
**91**	648	1.488	1.646	1.763	1.850

[a] $\beta = 100$ Hartree^{-1} and $N_{MO} = 100$.
[b] $\beta = 100$ Hartree^{-1} and $N_{MO} = 60$.
[c] $\beta = 100$ Hartree^{-1} and $N_{MO} = 80$.

Table S15: Emission wavelengths (λ_{em}, nm), emission energies (E_{em}, eV) and theoretically computed E_{S_1} (eV) values of boron(III)-based TADF emitters.

	$\lambda_{em}{}^a$	$E_{em}{}^a$	$E_{S_1,ZINDO/S}$	$E_{S_1,xTB-sTDA}$	$E_{S_1,rVGG}$	$E_{em,cal}{}^b$	Error^c
**4**	537	2.309	3.129	3.330	2.598	2.309	0.000
**21**	468	2.649	3.127	3.367	3.073	2.649	0.000
**22**	522	2.375	3.128	3.333	2.698	2.380	0.005
**93**	461	2.689	3.177	3.488	3.263	2.786	0.096
**94**	457	2.713	3.150	3.397	3.070	2.647	−0.066
**95**	469	2.644	3.128	3.365	3.075	2.651	0.007
**96**	481	2.578	3.096	3.349	2.996	2.594	0.017
**97**	472	2.627	3.104	3.350	3.185	2.730	0.103
**98**	520	2.384	3.032	3.376	2.775	2.436	0.051
**99**	506	2.450	3.135	3.406	2.957	2.566	0.116
**100**	488	2.541	3.197	3.425	2.929	2.546	0.006
MAE							0.052^d

^{a}Measured in aerated toluene solution with a concentration of 10^{-5} M.[3,18]
^{b}Calibrated using **4** and **21**, $E_{em,cal} = (E_{S_1,rVGG} + 0.6224)/1.3948$.
^{c}Error = $E_{em,cal} - E_{em}$.
^{d}Compounds **4** and **21** are excluded in the computation of MAE (eV).

Table S16: Wall times (s) required to generate the orbitals (ψ) and $\epsilon V^{\mathrm{H}} V^{\mathrm{P}}$ MolOrbImages for the YAM100 and QM9-ES data sets. All simulations are performed using 16 OpenMP threads on the computing clusters offered by Information Technology Services, The University of Hong Kong.

		SAD[a]		GFN2-xTB		PBE0[a]	
		ψ	$\epsilon V^{\mathrm{H}} V^{\mathrm{P}}$	ψ	$\epsilon V^{\mathrm{H}} V^{\mathrm{P}}$	ψ	$\epsilon V^{\mathrm{H}} V^{\mathrm{P}}$
YAM100	Simulation 1	4,242	13,187	156	4,038	114,114	13,051
	Simulation 2	3,695	17,658	111	4,775	141,066	17,435
	Simulation 3	7,737	19,795	142	5,253	178,482	19,404
	Simulation 4	4,276	19,470	145	5,131	146,466	18,136
QM9-ES	Simulation 1	229	1,273	27	189	6,958	1,320
	Simulation 2	343	1,299	43	240	7,160	1,304
	Simulation 3	404	1,406	35	261	7,934	1,470
	Simulation 4	330	1,417	29	226	7,786	1,427

[a]Def2-SVP and cc-pVTZ are used for the YAM100 and QM9-ES data sets, respectively.

Supplementary figures

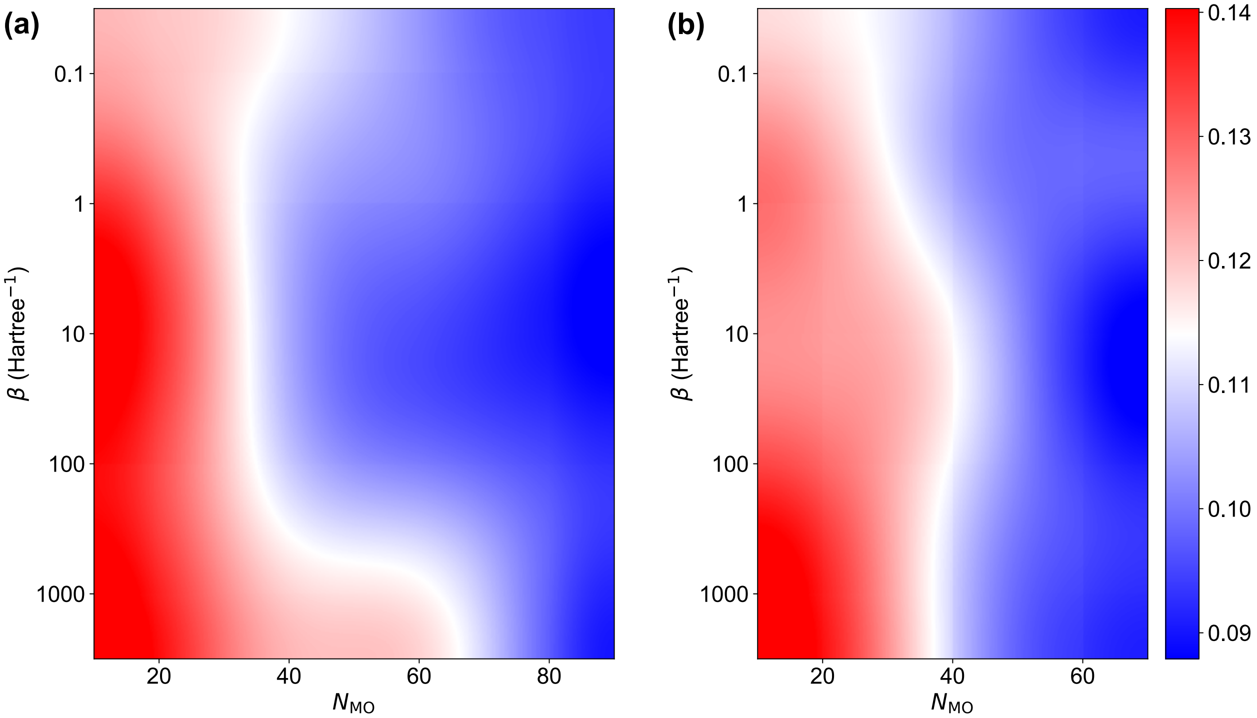

Figure S1: L_1 deviations (eV) of the (a) rVGG(SAD) and (b) rVGG(GFN2-xTB) predictions as a function of β and N_{MO} for QM9-40K data set.

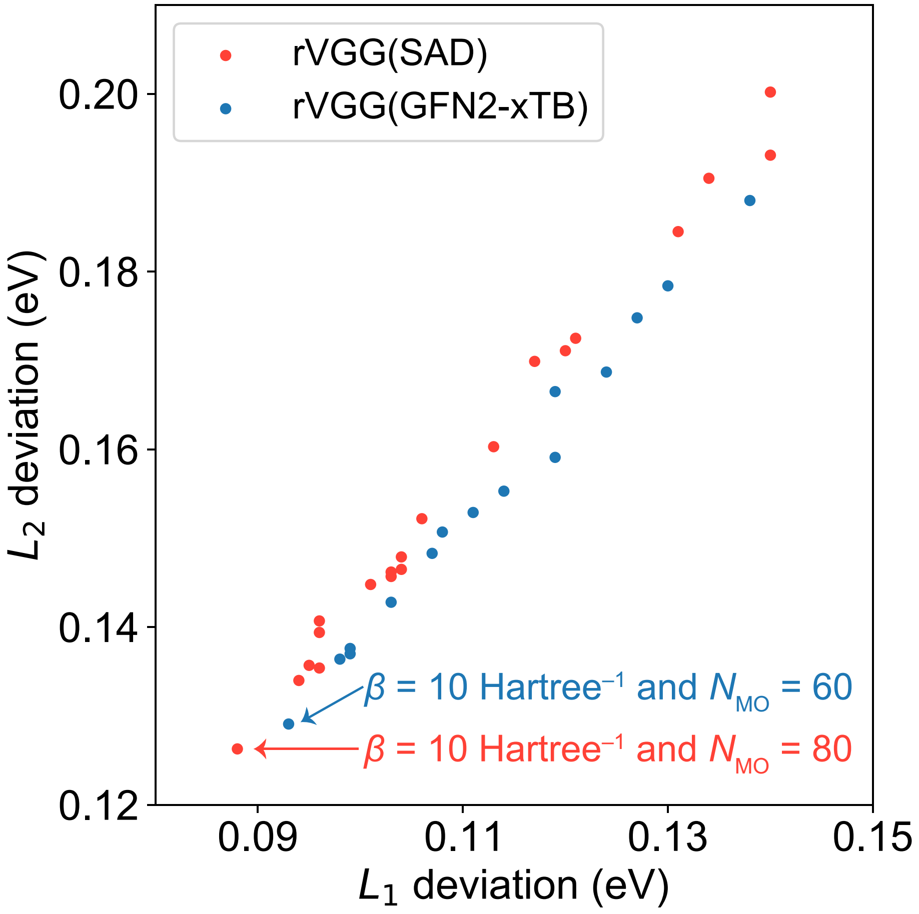

Figure S2: Correlation between the L_1 and L_2 deviations for the QM9-40K data set. Here, $L_2 = \sqrt{\frac{1}{6N_{\text{test}}}\sum_n^3\sum_i^{N_{\text{test}}}\left|E_{\text{S}_n}^{\text{rVGG}}(i) - E_{\text{S}_n}^{\text{ref}}(i)\right|^2 + \left|E_{\text{T}_n}^{\text{rVGG}}(i) - E_{\text{T}_n}^{\text{ref}}(i)\right|^2}$. It is clear that the model with the lowest L_1 deviation also yields the lowest L_2 deviation.

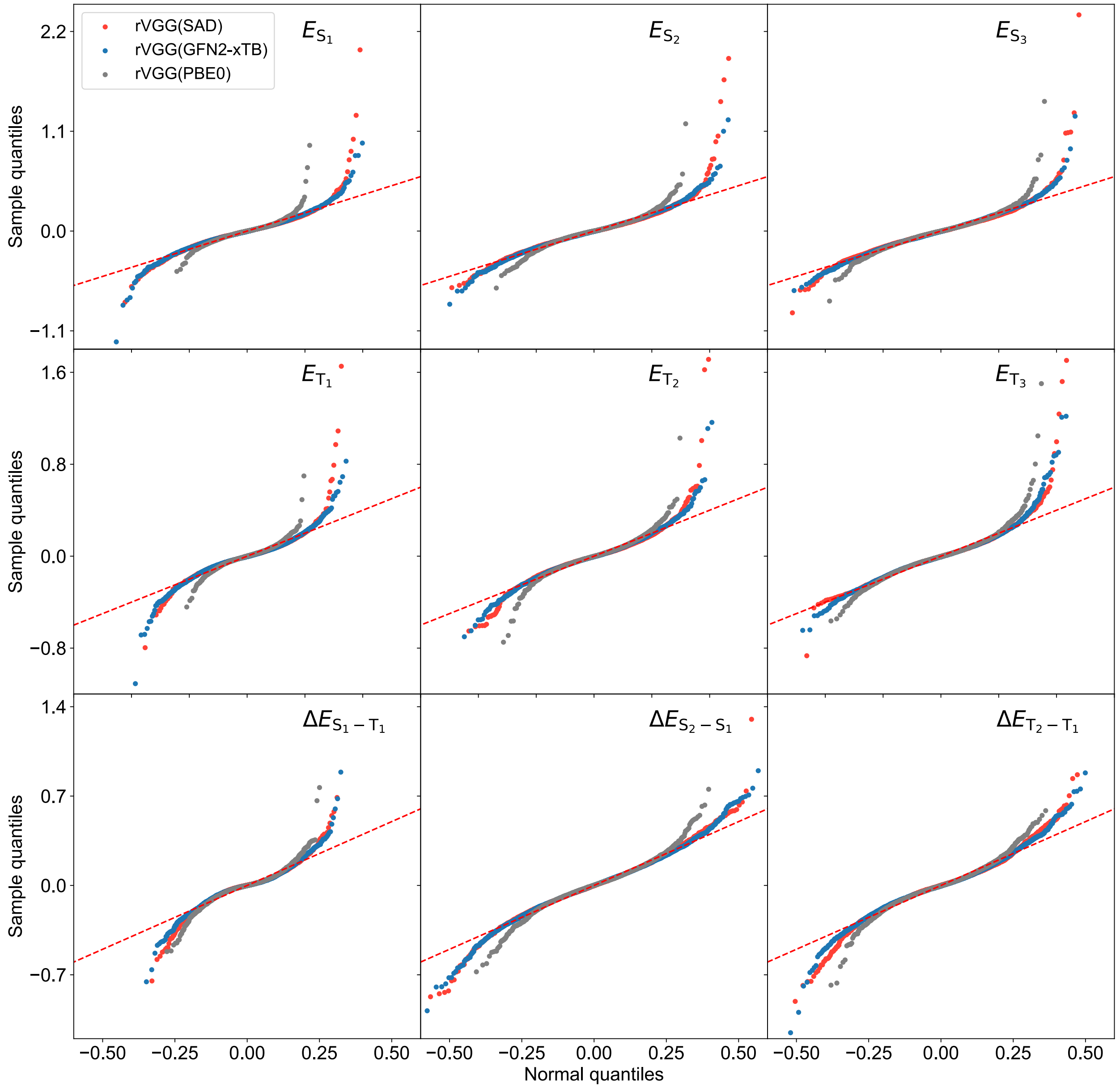

Figure S3: Quantile−quantile plots of errors (eV) in predicting excited-state properties computed at the ADC(2)/cc-pVTZ level for the QM9-40K data set.

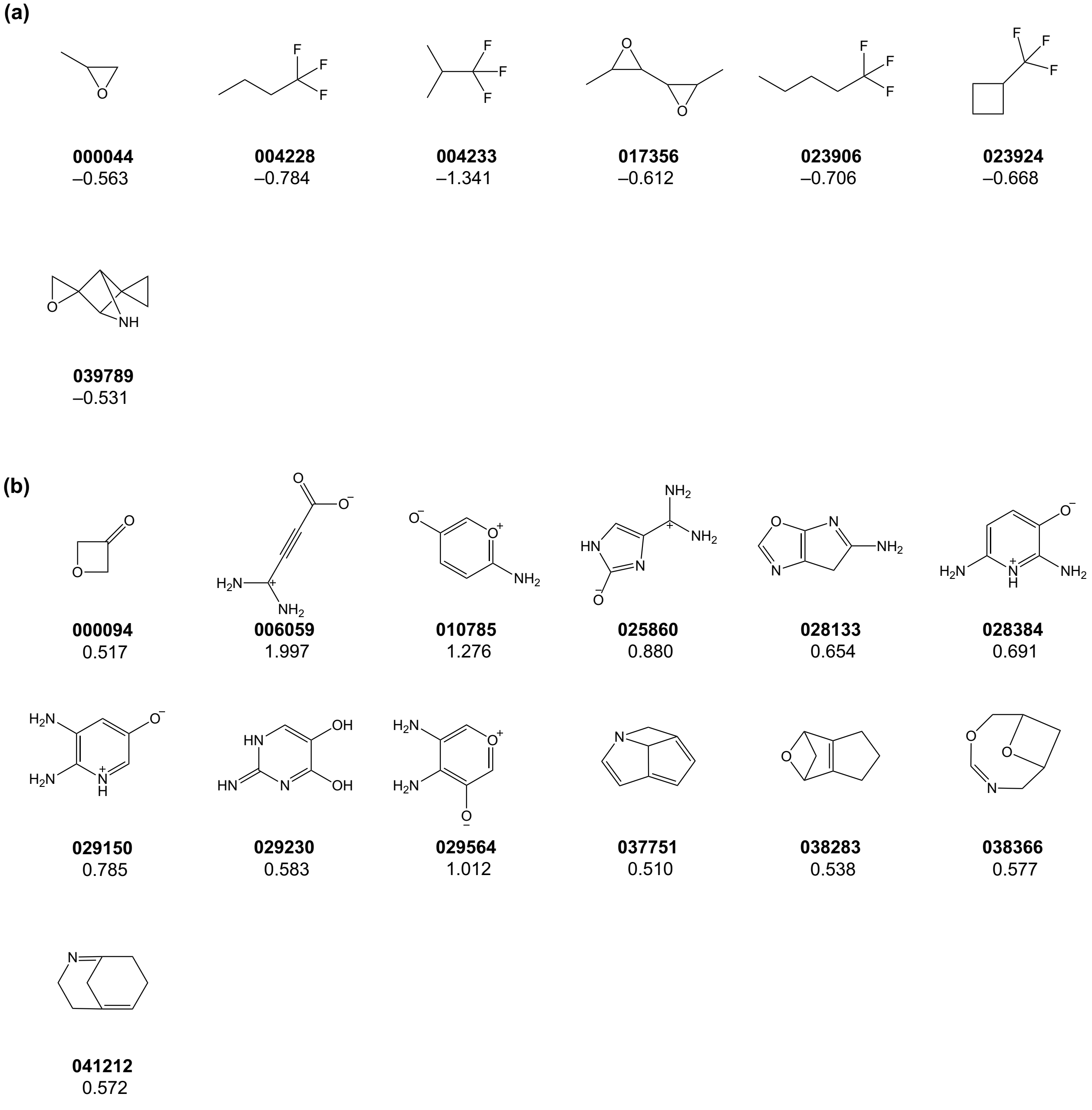

Figure S4: Molecular structures of extreme outliers for the optimal rVGG(SAD) model with (a) $E_{S_1}^{\text{rVGG}} - E_{S_1}^{\text{ref}} < -0.5$ eV and (b) $E_{S_1}^{\text{rVGG}} - E_{S_1}^{\text{ref}} > 0.5$ eV in predicting E_{S_1} for the QM9-40K data set. Indices (bold) in the QM9-40K data set and prediction errors (eV) are provided. Here, $\beta = 10$ Hartree^{-1} and $N_{\text{MO}} = 80$.

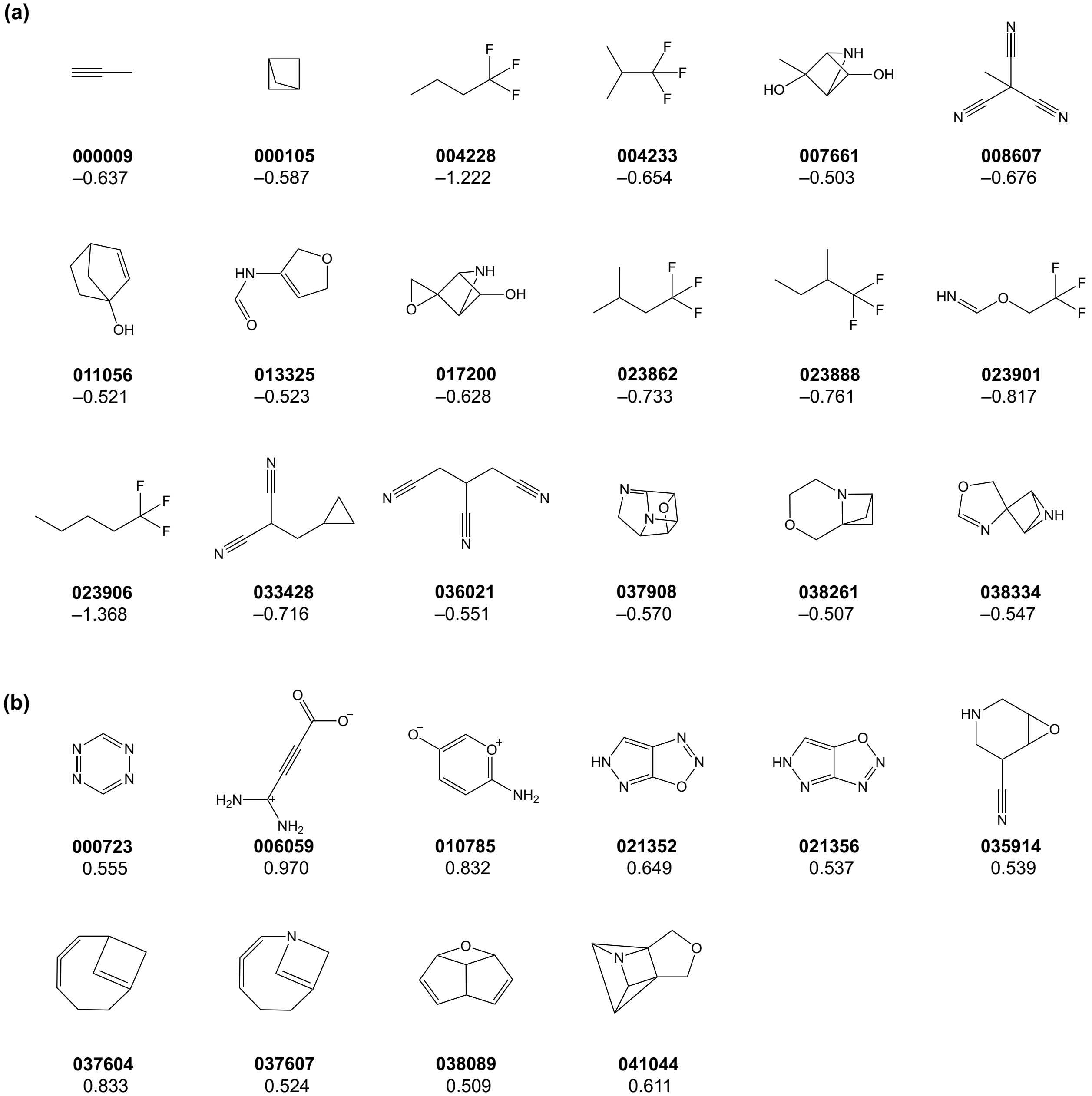

Figure S5: Molecular structures of extreme outliers for the optimal rVGG(GFN2-xTB) model with (a) $E_{\mathrm{S_1}}^{\mathrm{rVGG}} - E_{\mathrm{S_1}}^{\mathrm{ref}} < -0.5$ eV and (b) $E_{\mathrm{S_1}}^{\mathrm{rVGG}} - E_{\mathrm{S_1}}^{\mathrm{ref}} > 0.5$ eV in predicting $E_{\mathrm{S_1}}$ for the QM9-40K data set. Indices (bold) in the QM9-40K data set and prediction errors (eV) are provided. Here, $\beta = 10$ Hartree^{-1} and $N_{\mathrm{MO}} = 60$.

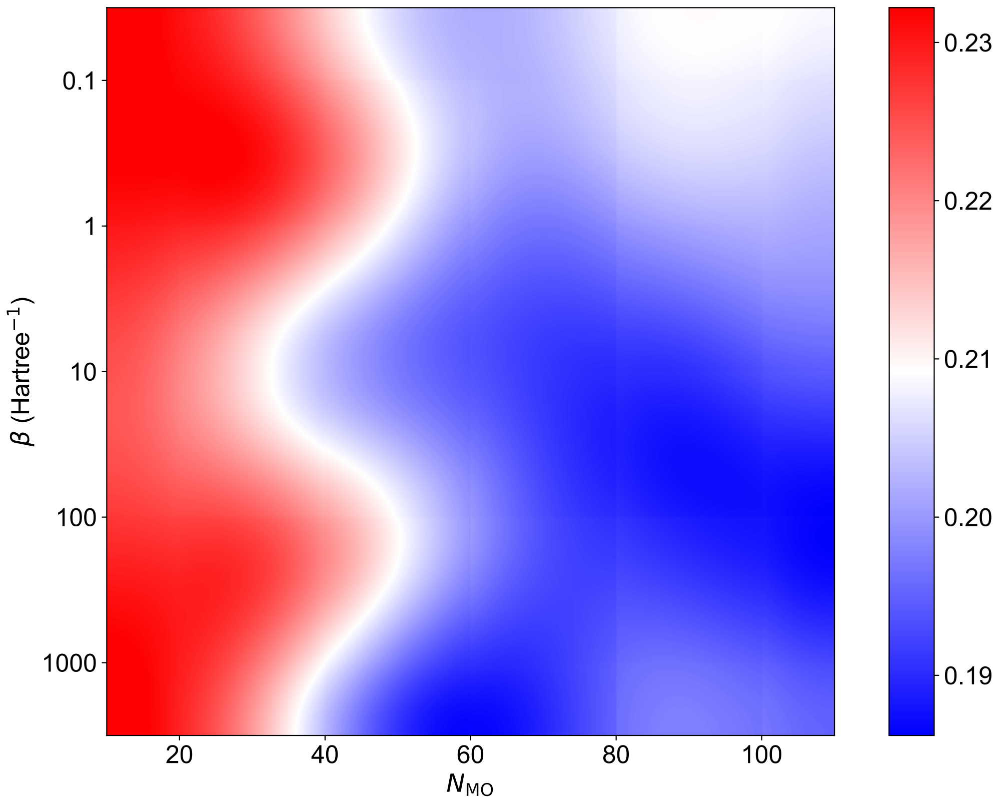

Figure S6: L_1 deviations (eV) of the rVGG(SAD) prediction as a function of β and N_{MO} for the CSD data set.

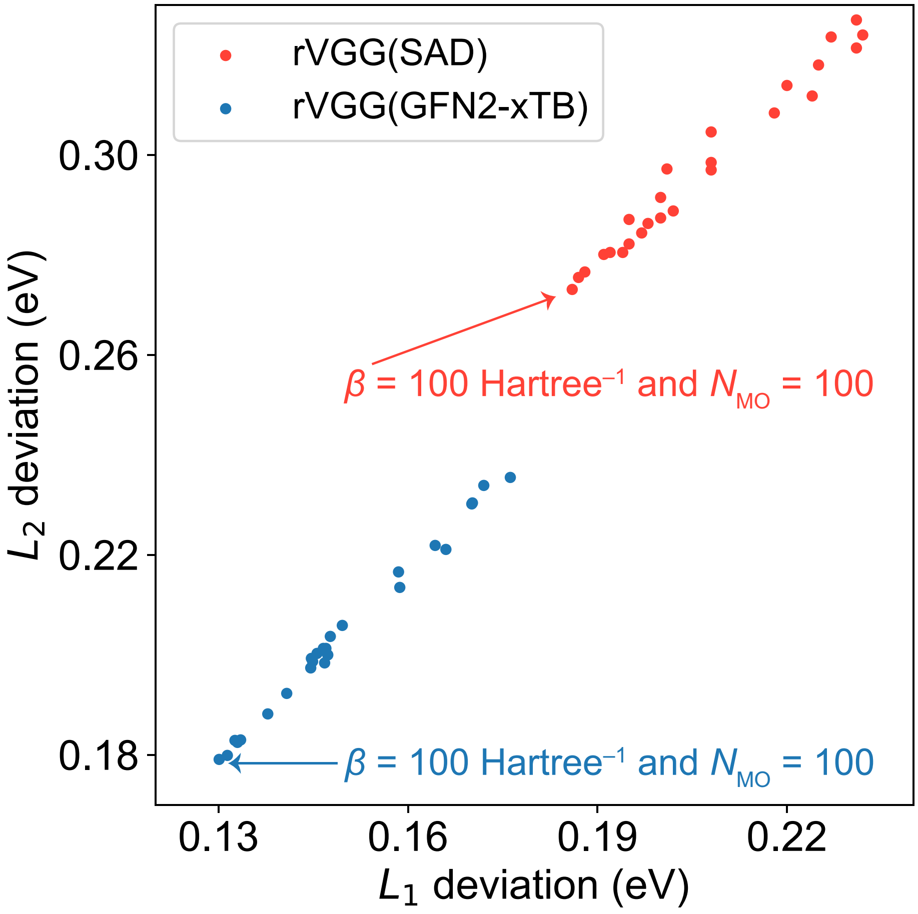

Figure S7: Correlation between the L_1 and L_2 deviations for the CSD data set. The model with the lowest L_1 deviation also yields the lowest L_2 deviation.

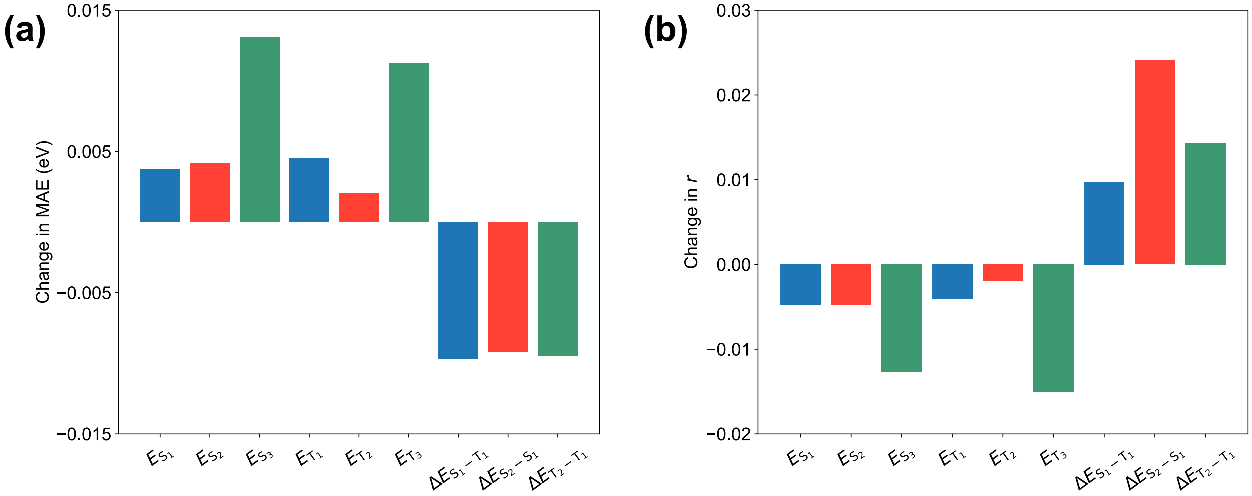

Figure S8: Change in (a) MAE and (b) r of excited-state properties predicted using the rVGG(GFN2-xTB) model upon utilization of a combined RMSE loss function on both excited-state energies (loss weight = 1) and gaps (loss weight = 20).

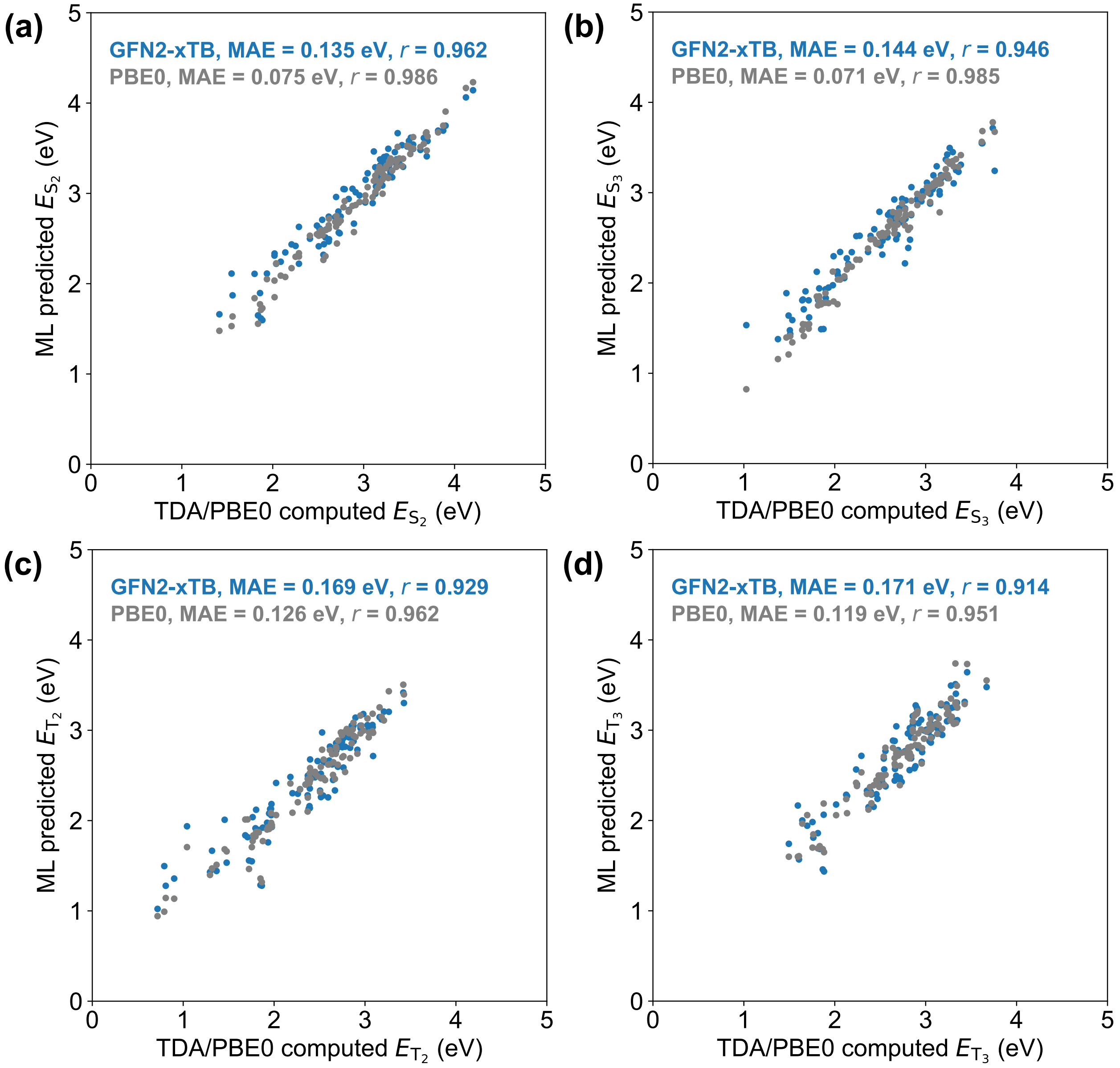

Figure S9: Correlation between the rVGG prediction and TDA/PBE0 computed reference for (a) E_{S_2}, (b) E_{S_3}, (c) E_{T_2} and (d) E_{T_3} for 100 organic photofunctional materials in the YAM100 data set.

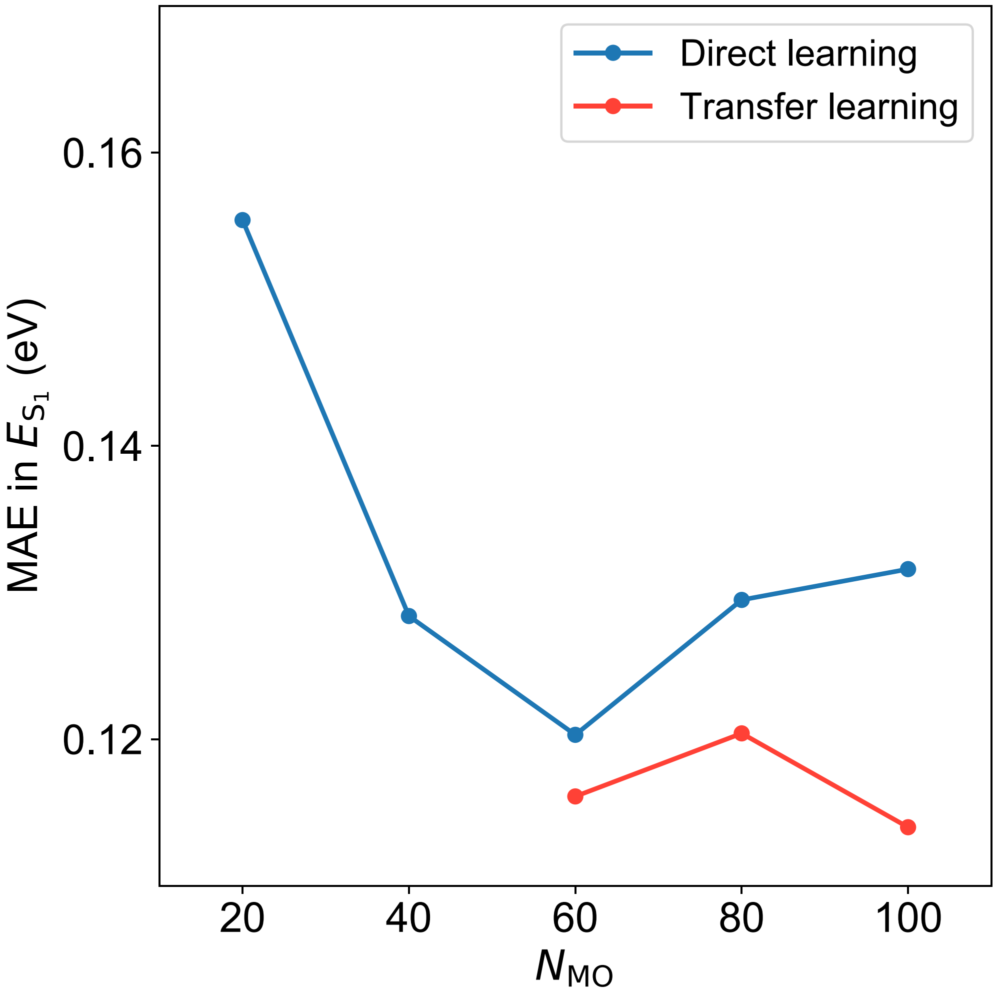

Figure S10: MAE in E_{S_1} as a function of N_{MO} of the rVGG(GFN2-xTB) model for 100 organic photofunctional materials in the YAM100 data set. With the direct learning, the elevated MAE values for $N_{MO} > 60$ indicate an increased risk of overfitting. The application of transfer learning strategy partially mitigates the overfitting issue. Here, $\beta = 100$ Hartree^{-1}.

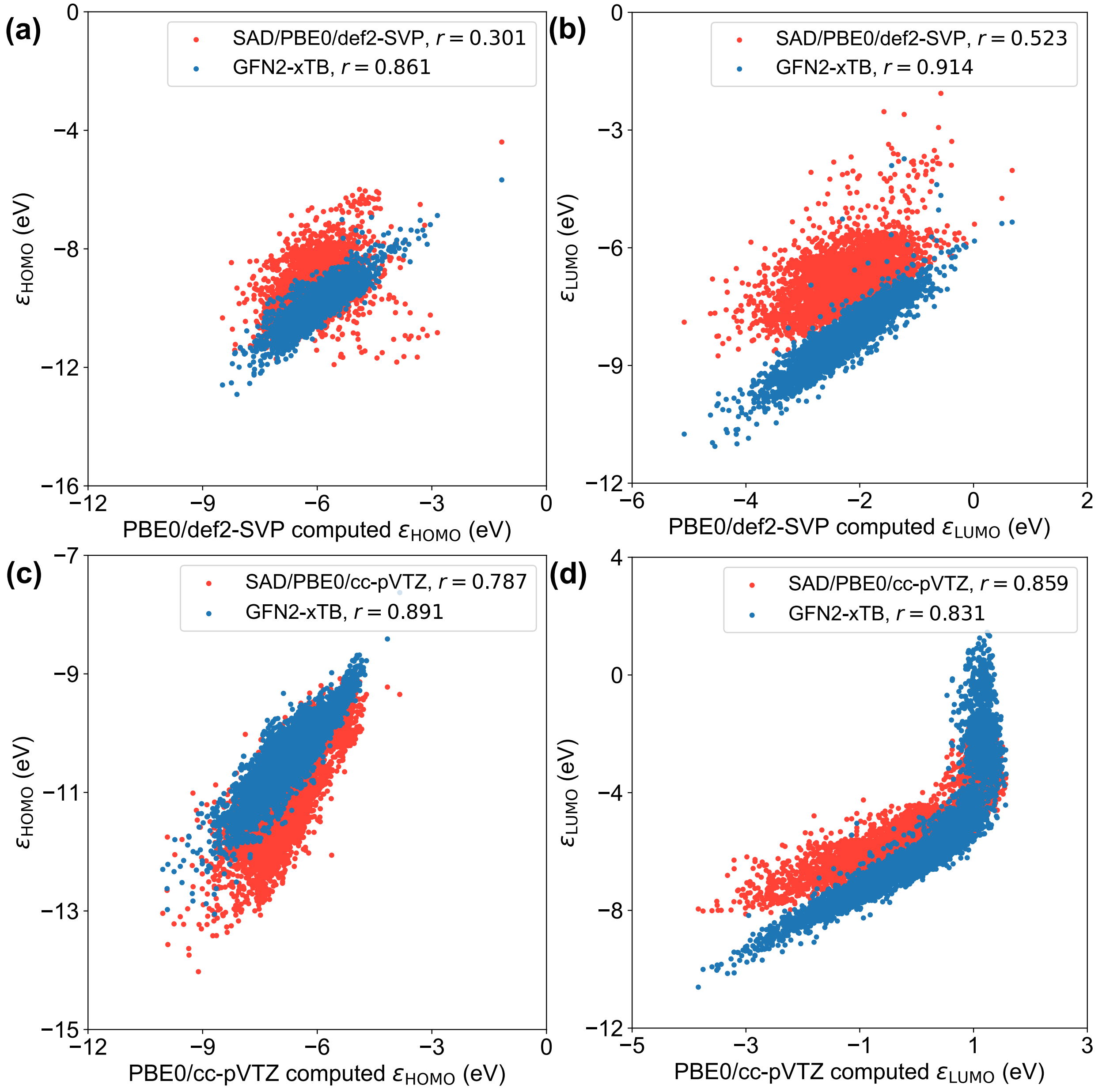

Figure S11: Correlations among SAD, GFN2-xTB and PBE0 computed ϵ_{HOMO} and ϵ_{LUMO} for the (a, b) CSD and (c, d) QM9-40K data sets.

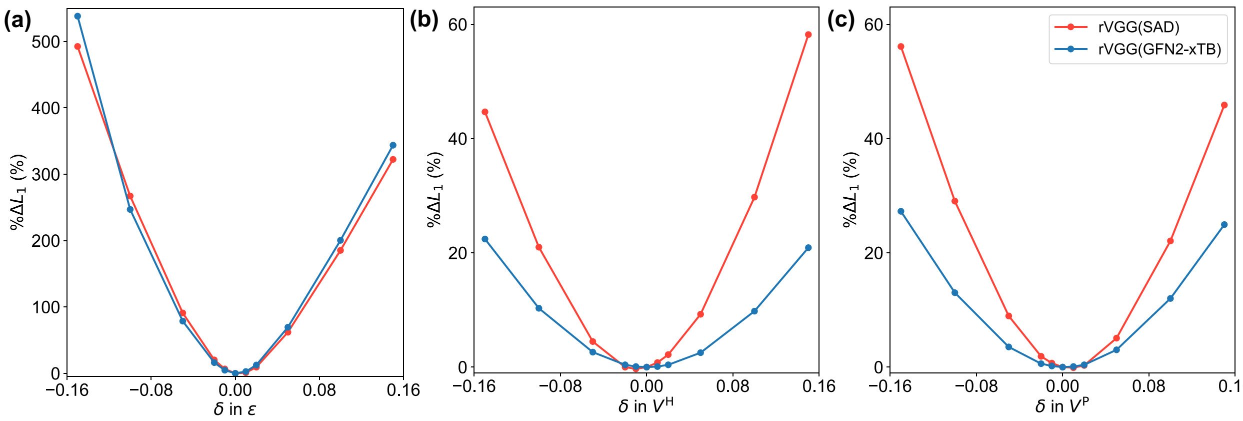

Figure S12: $\%\Delta L_1$ resulting from (a) ϵ, (b) V^{H} and (c) V^{P} channel-wise perturbations applied to $\epsilon V^{\mathrm{H}} V^{\mathrm{P}}$ for the QM9-40K data set.

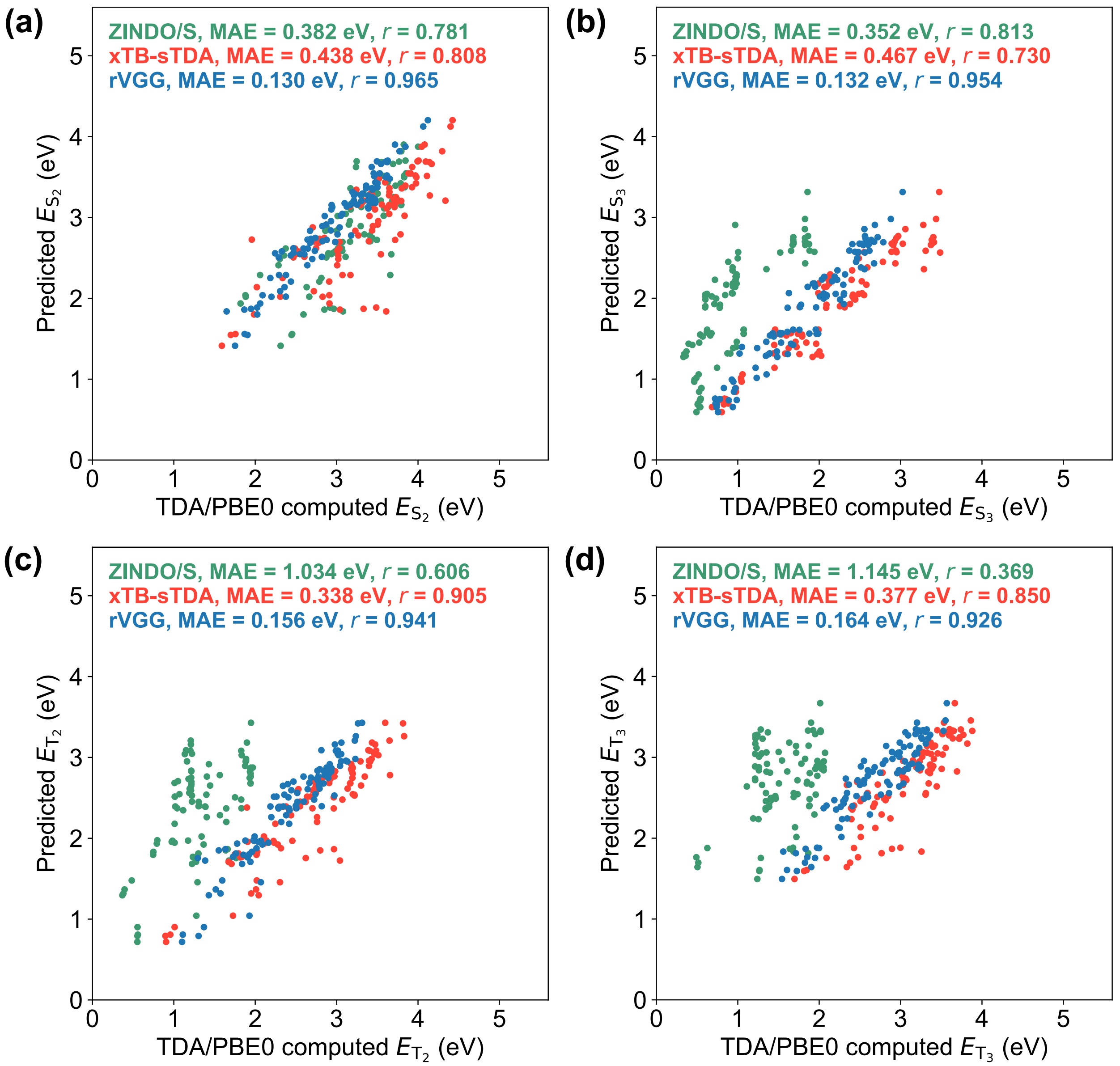

Figure S13: Theoretical predictions from ZINDO/S, xTB-sTDA and rVGG approaches are compared with TDA/PBE0 computed reference for (a) E_{S_2}, (b) E_{S_3}, (c) E_{T_2} and (d) E_{T_3} of 100 organic photofunctional materials in the YAM100 data set.

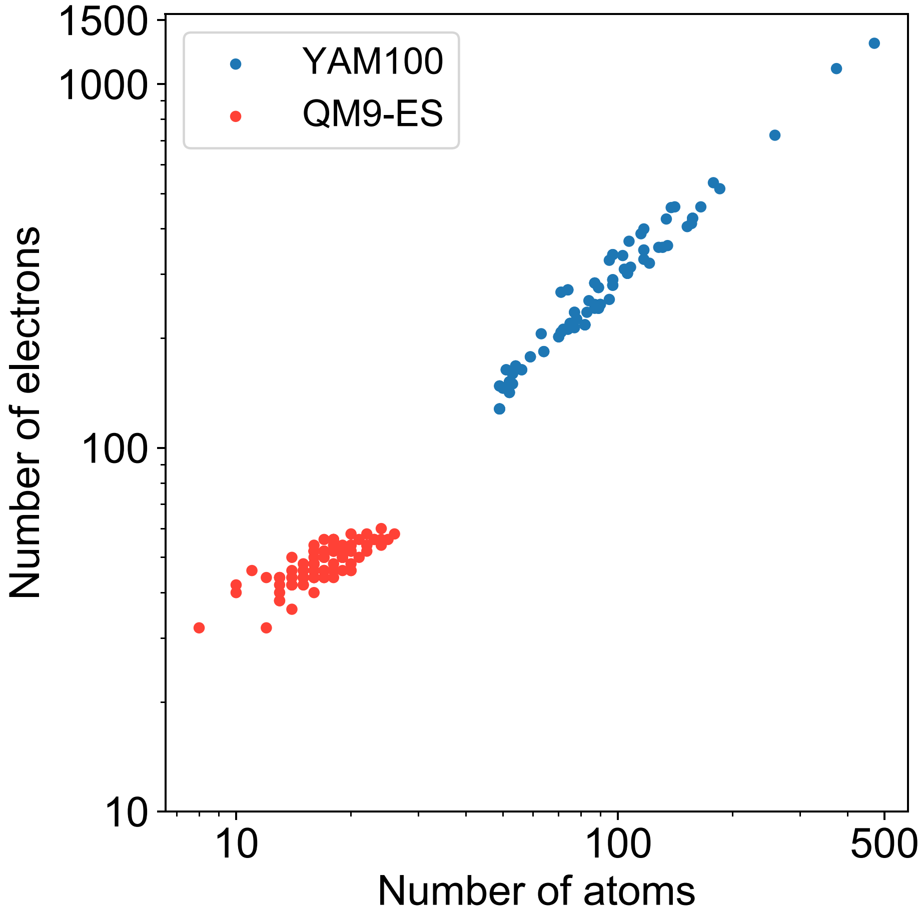

Figure S14: Comparison of the molecular size between the YAM100 and QM9-ES data sets.

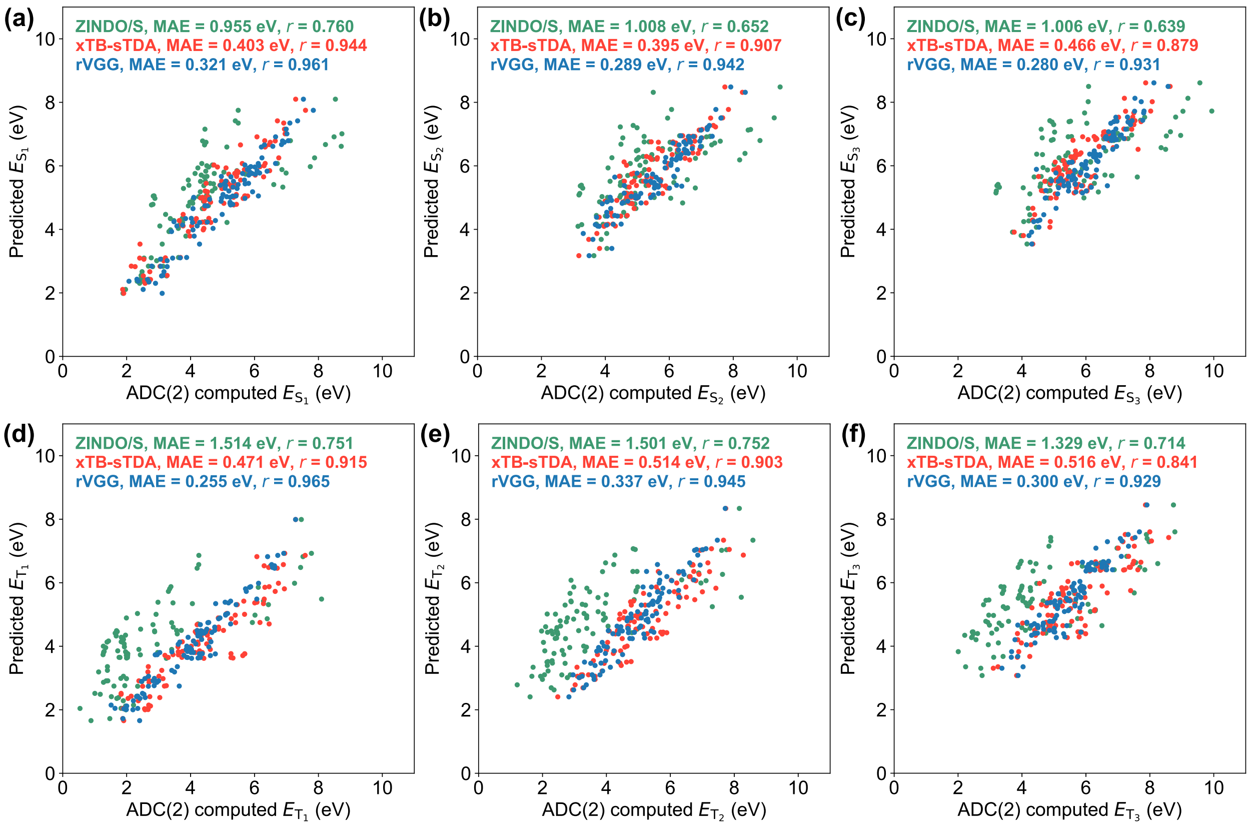

Figure S15: Correlation between the rVGG prediction and ADC(2) computed reference for (a) E_{S_1}, (b) E_{S_2}, (c) E_{S_3}, (d) E_{T_1}, (e) E_{T_2} and (f) E_{T_3} for molecules in the QM9-ES data set.

References

(1) Wu, N. M.-W.; Ng, M.; Lam, W. H.; Wong, H.-L.; Yam, V. W.-W. Photochromic Heterocycle-Fused Thieno[3,2-*b*]phosphole Oxides as Visible Light Switches without Sacrificing Photoswitching Efficiency. *J. Am. Chem. Soc.* **2017**, *139*, 15142–15150.

(2) Xu, Y.; Steudel, F.; Leung, M.-Y.; Xia, B.; von Delius, M.; Yam, V. W.-W. [*n*]Cycloparaphenylene-Pillar[5]arene Bismacrocycles: Their Circularly Polarized Luminescence and Multiple Guest Recognition Properties. *Angew. Chem. Int. Ed.* **2023**, *62*, e202302978.

(3) Li, P.; Chan, H.; Lai, S.-L.; Ng, M.; Chan, M.-Y.; Yam, V. W.-W. Four-Coordinate Boron Emitters with Tridentate Chelating Ligand for Efficient and Stable Thermally Activated Delayed Fluorescence Organic Light-Emitting Devices. *Angew. Chem. Int. Ed.* **2019**, *58*, 9088–9094.

(4) Chan, C.-Y.; Wong, Y.-C.; Chan, M.-Y.; Cheung, S.-H.; So, S.-K.; Yam, V. W.-W. Hole-Transporting Spirothioxanthene Derivatives as Donor Materials for Efficient Small-Molecule-Based Organic Photovoltaic Devices. *Chem. Mater.* **2014**, *26*, 6585–6594.

(5) Chan, C.-Y.; Wong, Y.-C.; Chan, M.-Y.; Cheung, S.-H.; So, S.-K.; Yam, V. W.-W. Bifunctional Heterocyclic Spiro Derivatives for Organic Optoelectronic Devices. *ACS Appl. Mater. Interfaces* **2016**, *8*, 24782–24792.

(6) Li, P.; Chan, C.-Y.; Lai, S.-L.; Chan, H.; Leung, M.-Y.; Hong, E. Y.-H.; Li, J.; Wu, H.; Chan, M.-Y.; Yam, V. W.-W. Three-Dimensional Spirothienoquinoline-Based Small Molecules for Organic Photovoltaic and Organic Resistive Memory Applications. *ACS Appl. Mater. Interfaces* **2020**, *12*, 11865–11875.

(7) Li, P.; Liang, Q.; Hong, E. Y.-H.; Chan, C.-Y.; Cheng, Y.-H.; Leung, M.-Y.; Chan, M.-Y.; Low, K.-H.; Wu, H.; Yam, V. W.-W. Boron(III) β-Diketonate-Based Small

Molecules for Functional Non-Fullerene Polymer Solar Cells and Organic Resistive Memory Devices. *Chem. Sci.* **2020**, *11*, 11601–11612.

(8) Li, Z.; Hong, E. Y.-H.; Poon, C.-T.; Cheng, Y.-H.; Chan, M. H.-Y.; Leung, M.-Y.; Wu, L.; Yam, V. W.-W. Synthesis, Characterization, Supramolecular Self-Assembly, and Organic Resistive Memory Applications of BODIPY Derivatives. *ACS Materials Lett.* **2023**, *5*, 909–919.

(9) Cheng, Y.-H.; Wong, H.-L.; Hong, E. Y.-H.; Leung, M.-Y.; Lai, S.-L.; Yam, V. W.-W. Design and Synthesis of Solution-Processable Donor−Acceptor Dithienophosphole Oxide Derivatives for Multilevel Organic Resistive Memories. *ACS Materials Lett.* **2020**, *2*, 1590–1597.

(10) Wu, N. M.-W.; Fung, T. H.-C.; Ng, M.; Yam, V. W.-W. Benzo[*b*]Germole-Fused Diarylethenes as Photochromic Organogermanium Compounds. *ACS Materials Lett.* **2024**, *6*, 314–320.

(11) Cheng, Y.-H.; Wong, H.-L.; Hong, E. Y.-H.; Lai, S.-L.; Chan, M.-Y.; Yam, V. W.-W. Versatile Phosphole Derivatives with Photovoltaic, Light-Emitting, and Resistive Memory Properties. *ACS Appl. Energy Mater.* **2020**, *3*, 3059–3070.

(12) Fung, T. H.-C.; Wong, C.-L.; Tang, W. K.; Leung, M.-Y.; Low, K.-H.; Yam, V. W.-W. Photochromic Dithienylethene-Containing Four-Coordinate Boron(III) Compounds with a Spirocyclic Scaffold. *Chem. Commun.* **2022**, *58*, 4231–4234.

(13) Poon, C.-T.; Lam, W. H.; Yam, V. W.-W. Gated Photochromism in Triarylborane-Containing Dithienylethenes: A New Approach to a "Lock-Unlock" System. *J. Am. Chem. Soc.* **2011**, *133*, 19622–19625.

(14) Yin, Z.; Tam, A. Y.-Y.; Wong, K. M.-C.; Tao, C.-H.; Li, B.; Poon, C.-T.; Wu, L.; Yam, V. W.-W. Functionalized BODIPY with Various Sensory Units − A Versatile

Colorimetric and Luminescent Probe for pH and Ions. *Dalton Trans.* **2012**, *41*, 11340–11350.

(15) Chan, J. C.-H.; Lam, W. H.; Yam, V. W.-W. A Highly Efficient Silole-Containing Dithienylethene with Excellent Thermal Stability and Fatigue Resistance: A Promising Candidate for Optical Memory Storage Materials. *J. Am. Chem. Soc.* **2014**, *136*, 16994–16997.

(16) Poon, C.-T.; Lam, W. H.; Yam, V. W.-W. Synthesis, Photochromic, and Computational Studies of Dithienylethene-Containing β-Diketonate Derivatives and Their Near-Infrared Photochromic Behavior Upon Coordination of a Boron(III) Center. *Chem. Eur. J.* **2013**, *19*, 3467–3476.

(17) Chan, C.-Y.; Wong, Y.-C.; Wong, H.-L.; Chan, M.-Y.; Yam, V. W.-W. A New Class of Three-Dimensional, p-Type, Spirobifluorene-Modified Perylene Diimide Derivatives for Small Molecular-Based Bulk Heterojunction Organic Photovoltaic Devices. *J. Mater. Chem. C* **2014**, *2*, 7656–7665.

(18) Li, P.; Lai, S.-L.; Chen, Z.; Tang, W. K.; Leung, M.-Y.; Ng, M.; Kwok, W.-K.; Chan, M.-Y.; Yam, V. W.-W. Achieving Efficient and Stable Blue Thermally Activated Delayed Fluorescence Organic Light-Emitting Diodes Based on Four-Coordinate Fluoroboron Emitters by Simple Substitution Molecular Engineering. *Chem. Sci.* **2024**, *15*, 12606–12615.

(19) Chen, Z.; Yam, V. W.-W. Encoding Hole−Particle Information in the Multi-Channel MolOrbImage for Machine-Learned Excited-State Energies of Large Photofunctional Materials. *J. Am. Chem. Soc.* **2023**, *145*, 24098–24107.

(20) Chen, Z.; Yam, V. W.-W. Machine-Learned Electronically Excited States with the MolOrbImage Generated from the Molecular Ground State. *J. Phys. Chem. Lett.* **2023**, *14*, 1955–1961.
